# Ultrafast Chemical Pre‐Sodiation of Pseudocapacitive CaV_8_O_20_ Anodes for High‐Performance Sodium‐Ion Capacitors

**DOI:** 10.1002/advs.77176

**Published:** 2026-08-18

**Authors:** Neetu Bansal, Anwar Hussain, Radhey Shyam Yadav, Prakash Kumar Pathak, Minjoong Kim, Tobias Mattisson, Heejoon Ahn, Rahul R. Salunkhe

**Affiliations:** ^1^ Materials Research Laboratory Department of Physics Indian Institute of Technology (IIT) Jammu Jammu J&K India; ^2^ Department of Environmental and Energy Sciences Division of Energy Technology Chalmers University of Technology Gothenburg Sweden; ^3^ Human‐Tech Convergence Program Department of Organic and Nano Engineering Hanyang University Seoul Republic of Korea; ^4^ Department of Battery Engineering Hanyang University Seoul Republic of Korea; ^5^ Department of Interdisciplinary Programs Indian Institute of Technology (IIT) Jammu Jammu J&K India

**Keywords:** calcium vanadate, chemical pre‐sodiation, intercalation pseudocapacitance, quasi‐zero‐strain mechanism, sodium‐ion capacitors, spectator effect

## Abstract

The practical implementation of sodium‐ion capacitors (SICs) is impeded by structural degradation of anode materials and irreversible depletion of active sodium during initial solid‐electrolyte interphase formation. Herein, we propose a synergistic design paradigm that combines a structurally robust calcium vanadate (CaV_8_O_20_) anode with a gas‐free, scalable chemical pre‐sodiation approach. Driven by the “spectator effect” of Ca^2+^ pillars, the pseudocapacitive CaV_8_O_20_ framework enables highly reversible, quasi‐zero‐strain Na^+^ transport, ensuring 90% capacity retention after 600 cycles in half‐cells. To overcome the initial active sodium losses, a rapid roll‐to‐roll chemical pre‐sodiation utilizing a highly reducing Na‐benzophenone solution is employed, yielding nearly 100% coulombic efficiency within just 2 min. Consequently, the pre‐sodiated full‐cell SIC delivers an exceptional specific energy of 176 Wh kg^−1^ and specific power of 380 W kg^−1^, alongside an outstanding lifespan of 10,000 cycles (87% retention). Coupled with stepwise operando electrochemical impedance spectroscopy to elucidate diffusion kinetics, this work establishes a scalable pathway to ultrastable, high‐performance SICs.

## Graphical abstract

An ultrafast (2‐minute), gas‐free chemical pre‐sodiation strategy using Na‐benzophenone is developed to compensate for initial sodium loss in calcium vanadate (CaV_8_O_20_). Driven by the Ca^2+^ ‘spectator effect’, the framework enables highly reversible, quasi‐zero‐strain solid‐solution sodium storage, explicitly bypassing destructive conversion reactions. Successfully translated to continuous roll‐to‐roll assembly, the pre‐sodiated sodium‐ion capacitor delivers 100% initial coulombic efficiency alongside exceptionally high energy and ultrastable cycling.

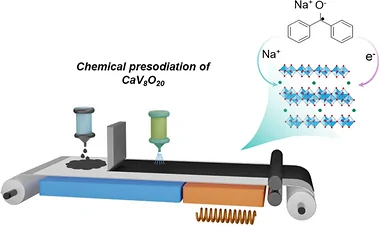

## 1 Introduction

Compared to lithium (Li), sodium (Na) reserves are nearly ten times higher, making Na‐ion technologies more cost‐effective and sustainable than Li‐ion batteries (LIBs) [1, 2]. However, traditional Na‐ion batteries (SIBs) often suffer from poor durability, primarily because the larger ionic radius of Na^+^ (0.106 nm) induces sluggish diffusion kinetics and accelerates progressive structural degradation of the electrode framework [3]. To circumvent these kinetic limitations, Na‐ion capacitors (SICs) elegantly bridge the gap between high‐energy batteries and high‐power capacitors by pairing a battery‐type anode with a capacitive cathode [4, 5, 6]. Thus, the ultimate commercialization of SICs hinges entirely on developing a robust battery‐type anode that can seamlessly match the ultrafast kinetics of the capacitive cathode without undergoing structural collapse.

Transition‐metal oxides, particularly vanadium‐based compounds, are promising anode candidates due to their multiple oxidation states, high specific capacity, low cost, and open‐framework structures that facilitate efficient Na^+^ storage [7, 8]. However, these vanadates are susceptible to severe volume expansion and metastable phase transitions during the continuous Na^+^ insertion and extraction, leading to rapid capacity fading [9]. To address this structural instability, metal pre‐intercalation presents a viable structural engineering strategy [10, 11, 12, 13]. These ions can enlarge the interplanar spacing and enable fast Na^+^ kinetics within the vanadate framework. Intriguingly, incorporating alkali metal ions, specifically Mg^2+^, Ca^2+^, and Sr^2+^, helps stabilize the structure through the “spectator effect” [14, 15, 16, 17]. In this mechanism, these ions function as electrochemically inactive structural pillars and do not participate in the redox reactions, thereby preventing framework collapse and enabling highly reversible, quasi‐zero‐strain Na^+^ transport [18, 19].

Despite achieving electrode stability in the anode, SICs still face a secondary, fatal flow: low initial coulombic efficiency (ICE). During the first cycle, the formation of the solid electrolyte interphase (SEI) layer irreversibly consumes active Na^+^ ions from the electrolyte, drastically reducing the energy density and cycle life of the assembled device. To mitigate this initial active Na loss, pre‐sodiation is essential. While various strategies exist, such as direct contact [20, 21], sacrificial salt addition [22], and electrochemical activation [23], they are fundamentally restricted by practical manufacturing limitations. Pre‐cycling for the electrochemical pre‐sodiation method requires tedious cell disassembly, and conventional sacrificial cathode additives require high‐voltage operation, leading to severe H_2_O/CO_2_/CO evolution that reduces sodiation efficiency, diminishes cell performance, and may cause swelling, raising safety concerns [24, 25]. In addition, direct contact pre‐sodiation is a simple and efficient method; however, it also poses significant safety concerns due to the high reactivity of Na metal. Consequently, there is an urgent need for an *ex situ*, gas‐free, and scalable pre‐sodiation technique.

Herein, we propose a synergistic strategy to realize ultrastable, high‐energy SICs by combining structurally stabilized calcium vanadate (CVO), specifically CaV_8_O_20_, as the anode with a laboratory‐scale roll‐to‐roll, compactible pre‐sodiation protocol. The pseudocapacitive CaV_8_O_20_ framework, stabilized by Ca^2+^ pillars, ensures highly reversible Na^+^ ion kinetics. To overcome the initial active Na loss, an ultrafast, roll‐to‐roll chemical pre‐sodiation utilizing a highly reducing Na‐benzophenone (Na‐BPh)/diglyme solution is employed. This approach drives rapid, spontaneous Na^+^ insertion, yielding ∼100% ICE within 2 min of soaking. The practical viability of the synergistic design was verified by preparing a pre‐sodiated CVO foil measuring 23 × 8 cm using the roll‐to‐roll method. When integrated with a porous carbon cathode, the fully assembled pre‐sodiated SIC delivers an exceptional specific energy of 176 Wh kg^−1^ at a specific power of 380 W kg^−1^. Furthermore, the device showcases an outstanding lifespan of 10,000 cycles with 87% capacity retention. Combined with stepwise operando electrochemical impedance spectroscopy (EIS) to elucidate the diffusion mechanism, this work establishes a scalable, structurally robust pathway for the next generation of high‐performance SICs.

## 2 Results and Discussion

### 2.1 Structural Evolution and the Dual Role of Confined Water

The pristine CVO (CVO*p*) was systematically synthesized by the hydrothermal method at 220°C (see the Experimental section for details). CVO*p* structure contains H_2_O molecules. While hydrated structure triggers parasitic reactions by the formation of NaOH during charging, which leads to excessive capacity loss [26, 27]. Therefore, to achieve optimal thermodynamic balance, removal of reactive intermediate species (‐H or ‐OH) is needed to facilitate rapid Na^+^ diffusion [28]. Thus, the CVO*p* sample was heat‐treated at 350°C, 450°C, and 550°C, and the resulting samples were designated CVO*350*, CVO*450*, and CVO*550*, respectively.

The powder X‐ray diffraction (PXRD) spectra of CVO*p* (Figure S1) crystallize in the pure monoclinic phase of CaV_8_O_20_·nH_2_O with C2/m space group (Ref. code: 00‐045‐1362). Featuring a layered structure containing Ca^2+^ ions and H_2_O molecules evenly distributed between the layers of V_8_O_20_ [29]. Thermal annealing at higher temperatures attenuates the peak intensities of CVO*350* (Figure S1) and CVO*450* (Figure 1a), reflecting diminished crystallinity of the samples [30]. Conversely, at 550°C, it triggers complete recrystallization (as evidenced by the PXRD peak intensity of CVO*550* in Figure 1a) via elevated atomic diffusion, effectively eliminating structural cavities and increasing lattice rigidity [16]. Crucially, the progressive removal of structural water induces a peak shift (Figure 1a; Figure S1) corresponding to the (001) reflection from 8.2° in CVO*p* to 8.5° in the annealed samples (CVO*350*‐*550*). Despite this minor contraction, the resulting interplanar spacing of 1.04 nm remains significantly larger than the Na^+^ ionic radius, preserving highly accessible 2D transport channels for rapid and facile ion intercalation. The inset of Figure 1a illustrates the crystal structure of CaV_8_O_20_·nH_2_O, featuring Ca^2+^ ions positioned between the edge‐shared VO_6_ polyhedral bilayers.

**FIGURE 1:**
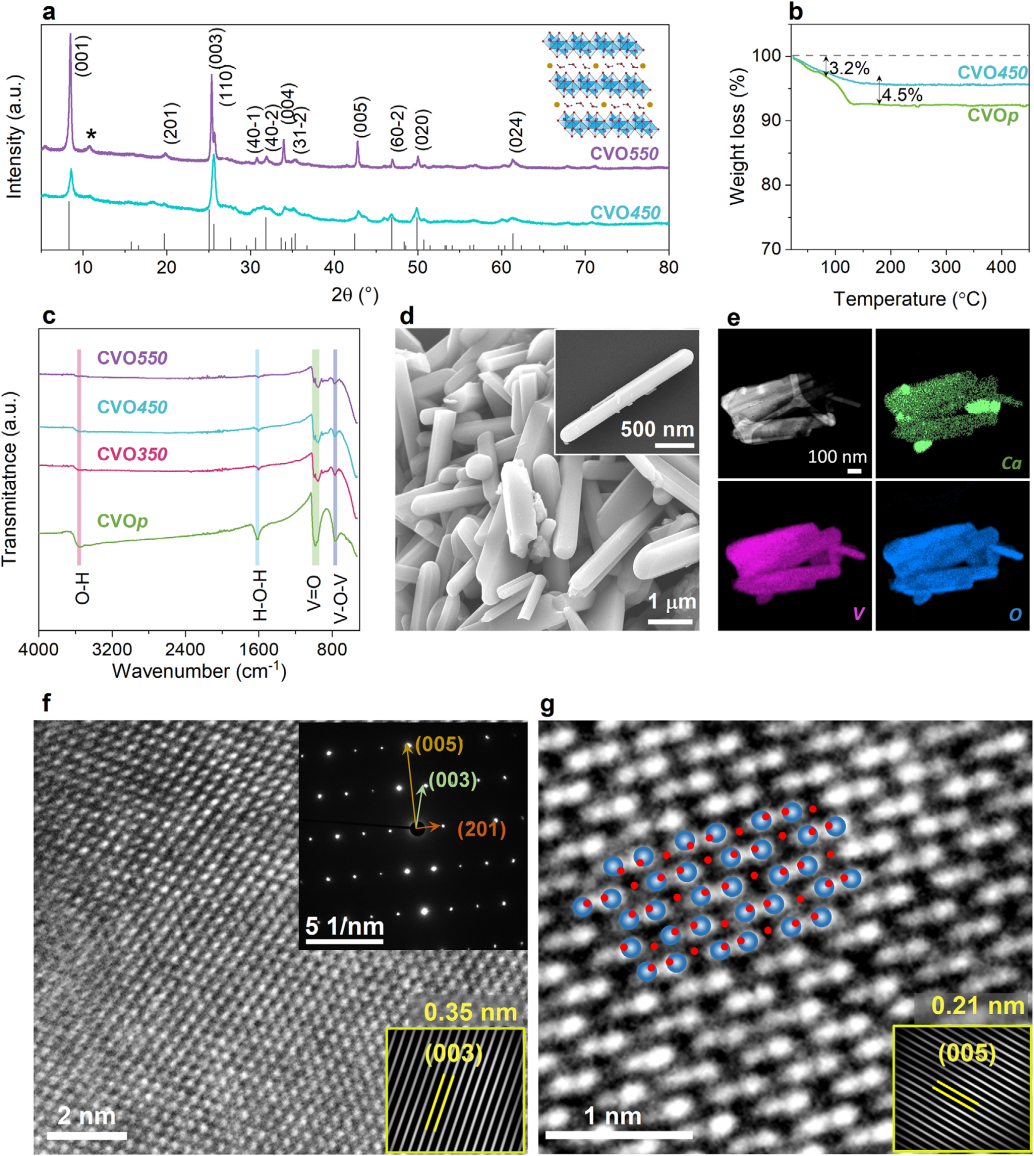
Physicochemical characterization of the different compositions of CVO materials. (a) PXRD spectra of CVO*450* and CVO*550* samples (inset: crystal structure of CVO*p*; blue spheres: V, red spheres: O, and yellow spheres: Ca). (b) TG curves for CVO*p* and CVO*450*. (c) FTIR spectra comparing the hydrated and dehydrated CVO samples. (d) FESEM image depicting the micro‐rod structure, with an inset showing a high‐magnification image of a single rod. (e) HAADF image of CVO and corresponding EDS mapping confirms the uniform presence of Ca, V, and O elements in the CVO*450* sample. (f) HRTEM image and SAED pattern displaying various CVO planes (003), (005), and (201). (g) HRTEM image and corresponding model for the CVO crystal structure (blue spheres: V, red spheres: O). Insets of (f) and (g) represent the *i*‐FFT patterns of respective HRTEM images.

The thermogravimetric analysis (TGA) curve (Figure 1b) quantitatively delineates the hydration state of the CVO*p* sample, exhibiting 3.2% loss below 100°C, attributed to physically adsorbed water, and 4.5% below 150°C, corresponding to interlayer‐confined water. The secondary mass loss corresponds to ∼2H_2_O molecules per unit cell, establishing the stoichiometric composition as CaV_8_O_20_·2H_2_O (calculations are described in Note S1). After water removal, CVO*450* and CVO*550* samples have a structural composition of CaV_8_O_20_. The progressive thermal dehydration is further corroborated by Fourier transform infrared (FTIR) spectroscopy (Figure 1c). The CVO*p* sample exhibits a broad band at ∼3560 cm^−1^, corresponding to O─H stretching vibrations of physically adsorbed and structurally bonded H_2_O molecules [31]. This peak is significantly weaker in CVO*350* and completely absent in CVO*450* and CVO*550*. Similarly, a small peak at 1610 cm^−1^ attributed to H─O─H bending vibrations progressively diminishes at higher annealing temperatures, consistent with the TGA results. Moreover, the characteristic vibrational modes at 765 cm^−1^ and 985 cm^−1^ are assigned to asymmetric V─O─V bridging and terminal V═O stretching vibrations, respectively, and remain robust across all annealing temperatures, confirming that the electrochemically active vanadate framework is perfectly preserved during the dehydration process [32].

Field‐emission scanning electron microscopy (FESEM) reveals the morphological evolution of the vanadate framework. While CVO*p* (Figure S2a) has a thin belt‐like morphology, with a length of a few microns, annealing at 350°C results in microbelts stacking on one another, forming thicker belts with distorted surfaces (Figure S2b). Water evaporation at elevated temperatures (as previously confirmed by TGA and FTIR analysis) is expected to form cavities, leading to a roughened surface [33]. Further, upon increasing the temperature to 450°C (CVO*450*), it triggers structural reorganization into well‐defined micro‐rods with relatively rounded boundaries (Figure 1d). Conversely, excessive dehydration in CVO*550* collapses the architecture into an irregular, needle‐like form (Figure S2c). The complete structural transition by annealing CVO*p* at different temperatures is shown schematically in Figure S3. For further discussion, only CVO*450* and CVO*550* with removed water samples are considered. Energy‐dispersive X‐ray spectroscopy (EDS) provided elemental mapping (Figure 1e) of the CVO*450* sample, revealing a uniform distribution of Ca, V, and O.

To probe the internal lattice integrity, high‐resolution transmission electron microscopy (HRTEM) and inverse fast Fourier transform (*i*‐FFT) analysis were conducted on CVO samples. HRTEM of CVO*450* revealed two distinct interplanar spacings of 0.35 nm (Figure 1f and inset) and 0.21 nm (Figure 1g and inset), aligned to (003) and (005) planes of the CaV_8_O_20_ structure, respectively. Crucially, the preservation of the expanded 0.35 nm (003) channels provides robust, low‐barrier 2D pathways for rapid Na^+^ diffusion, structurally supported by the intercalated Ca^2+^ pillars. The HRTEM image shown in Figure 1g was used to construct an approximate model of the CVO crystal structure by correlating it with the structural model derived from the PXRD studies. Here, only the atomic positions of vanadium are visible. The Ca and O are expected to have lower intensities and are therefore not identified in the HRTEM images. The diffraction spots corresponding to the (005), (003), and (201) planes can be observed in the selected‐area electron diffraction (SAED) pattern (inset of Figure 1f). HRTEM images at different magnifications for CVO*p* and CVO*550* are displayed in Figures S4 and S5, respectively, again showing similar structural properties with 0.35 nm spacing.

X‐ray photoelectron spectroscopy (XPS) further elucidates the interfacial chemical environment. The survey spectra (Figure S6) of CVO*p*, CVO*450*, and CVO*550* again confirm their purity, with peaks from Ca 2*p*, V 2*p*, and O 1*s* (C 1*s* due to adventitious carbon) elements. The Ca 2*p* deconvoluted spectra for CVO*450* and CVO*550* show peaks corresponding to Ca 2*p*_3/2_ and Ca 2*p*_1/2_ (Figure S7a,c). The V 2*p* spectra of CVO*450* and CVO*550* confirm the coexistence of mixed and valence states. This dual‐valence nature is highly advantageous for Na storage kinetics: V^5+^ serves as the primary redox center to maximize specific capacity, while V^4+^ provides partially filled 3*d* orbitals that intrinsically enhance the electronic conductivity of vanadates and interfacial charge‐transfer kinetics [34]. Notably, over‐annealing to 550°C induces the partial oxidation of V^4+^, severely diminishing this conductive advantage [28].

Finally, adsorption–desorption isotherms (N_2_, 77K) delineate the impact of these structural transformations on the specific surface area (SSA). The isotherms and corresponding pore‐size distributions for all samples are shown in Figure S8a,b. The Brunauer‐Emmett‐Teller (BET) multi‐point modeled SSA progressively increases from 11.4 m^2^ g^−1^ (CVO*p*) to 19.5 m^2^ g^−1^ (CVO*450*) and 33.2 m^2^ g^−1^ (CVO*550*) as water evaporation generates internal cavities (see Table S1 for complete details of SSA, average pore size, and pore volume). In this context, CVO*450* achieves an optimal kinetic balance: its surface area is sufficiently high to expose abundant redox‐active sites for ultrafast pseudocapacitive Na^+^ storage, yet appropriately restricted to suppress severe electrolyte decomposition and irreversible Na‐trapping.

### 2.2 Charge Storage Kinetics and “Quasi‐Zero‐Strain” Mechanism

To systematically evaluate the electrochemical behavior of the structurally optimized CVO framework, dehydrated CVO*450* and CVO*550* electrodes were assembled in the half‐cell configuration. During the galvanostatic charge‐discharge (GCD) at 0.05 A g^−1^, CVO*450* and CVO*550* delivered massive specific capacities of 811 and 839 mAh g^−1^, respectively (Figures 2a,b). The exceptional initial capacity is intrinsically driven by the multielectron redox capability of the highly active V^5+^ centers (V^5+^ ↔ V^4+^ ↔ V^3+^). The discharge capacities for the second to fifth cycles are 471 mAh g^−1^, 463 mAh g^−1^, 460 mAh g^−1^, and 453 mAh g^−1^ (44% capacity loss) for CVO*550*, and 519 mAh g^−1^, 513 mAh g^−1^, 514 mAh g^−1^, and 511 mAh g^−1^ (36% capacity loss) for CVO*450*. Following the irreversible consumption of active Na^+^ during the initial SEI, the reversible charge capacities stabilized at 493 mAh g^−1^ and 489 mAh g^−1^, respectively. More importantly, the macroscopic stability of CVO*450* and CVO*550* electrodes diverged significantly during the extended rate perforamce. The rate performance tests (Figure 2c,d) reveal that CVO*450* consistently outperforms CVO*550* at elevated current densities. The inferior stability of CVO*550* is a direct consequence of structural changes, which not only rigidify the lattice but also create an excessively high specific surface area that aggressively promotes continuous, unstable interfacial side reactions. In addition, the higher V^4+^/V^5+^ ratio in CVO*450* provides higher conductivity to the anode, thereby promoting the diffusion of ions [34, 35]. Conversely, CVO*450* maintains an optimal kinetic balance, in which Ca^2+^ ions act as robust, electrochemically inactive pillars. Because the 2+ oxidation state of calcium is highly stable within the 0.01–3.0 V operating window, these Ca^2+^ pillars restrict destructive volumetric changes without participating in the redox chemistry, a phenomenon known as the “spectator effect” [36, 37]. The complete GCD data (Figure 2e) show the stable performance of CVO*450* (>90% retention) for 600 cycles compared to CVO*550* (20% retention), and its performance outclasses many previous materials for SIBs, such as Co_2_VO_4_@C (87%/250 cycles) [38], WS_2_ (88%/500 cycles) [39], and Sb nanoflakes (59%/80 cycles) [40] (for more details, see Table S2). Thus, for further electrochemical performance, only the CVO*450* sample was selected.

**FIGURE 2:**
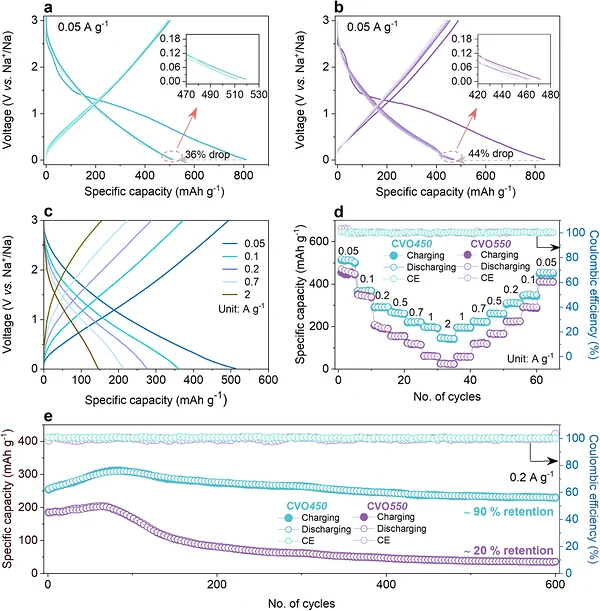
Electrochemical performance of half‐cells. GCD curves of (a) CVO*450* and (b) CVO*550* for the initial five cycles. An inset showing magnified GCD plots in the voltage range 0.01–0.2 V. (c) GCD plots for CVO*450* at various current rates. (d) Rate performance comparison between CVO*450* and CVO*550* half‐cells. (e) Cycle stability performance of both samples at 0.2 A g^−1^ over 600 cycles.

To quantify the speed and nature of charge storage within the stabilized CVO*450* framework, cyclic voltammetry (CV) was conducted over scan rates of 0.2–1.4 mV s^−1^ (Figure 3a,b). For the initial three CV cycles, the oxidation peak (1.02 V) shifts to lower voltages due to minute irreversible conversion reactions that occur during SEI layer formation (Figure 3a) [41]. Following the initial stabilization of the SEI layer, the CV curves maintained perfectly overlapping coordinates, thereby bypassing the continuous irreversible conversion reactions that typically plague unpillared transition‐metal oxides such as TiO_2_ or Nb_2_O_5_ [42, 43]. The peak currents corresponding to anodic and cathodic peaks at different scan rates are evaluated to yield a linear plot (Figure 3c) of log(peak current) vs. log(scan rate). Applying the power law relationship (Equation S1, Note S2), the calculated *b*‐values for the cathodic and anodic peaks were 0.84 and 0.83, respectively. This confirms that the Na^+^ storage in CVO*450* is overwhelmingly governed by surface‐controlled pseudocapacitance rather than sluggish semi‐infinite diffusion. Furthermore, capacitive and diffusive contributions were identified by employing Dunn's method at different sweep rates (Figure 3d). By deconvoluting the total current into capacitive (*k_1_v*) and diffusion‐controlled (*k_2_v* ^1/2^) contributions, we mathematically confirm that the capacitive charge storage fraction reaches an exceptional 91% at 1.4 mV s^−1^. Furthermore, Galvanostatic Intermittent Titration Technique (GITT) measurements (Equation S2 of Note S2) reveal striking Na^+^ diffusion coefficients (*D_Na_* ^+^) ranging from 10^−8^ cm^2^ s^−1^ to 10^−9^ cm^2^ s^−1^ (Figure 3e). This ultrafast ionic mobility, which significantly outpaces that of conventional carbonaceous (10^−9^–10^−10^ cm^2^ s^−1^) [44, 45] or chalcogenide‐based anodes (10^−10^–10^−12^ cm^2^ s^−1^) [44, 46]. It is directly enabled by the enlarged 0.35 nm interplanar spacing maintained by the Ca^2+^ pillars. Intriguingly, the strategically designed structure allows faster ionic movement compared to other vanadates utilizing Na, Ni and Ca pillars, such as Na_2_V_2_O_6_ (10^−10^ cm^2^ s^−1^ to 10^−13^ cm^2^ s^−1^) [47], Ni_2_VOH (10^−13^ cm^2^ s^−1^ to 10^−15^ cm^2^ s^−1^) [48], Na_2_CaV_2_O_6_F (10^−9^ cm^2^ s^−1^ to 10^−11^ cm^2^ s^−1^) [47], and HNa_0.4_V_6_O_16‐_ *_x_* ·3H_2_O (10^−10^ cm^2^ s^−1^) [48].

**FIGURE 3:**
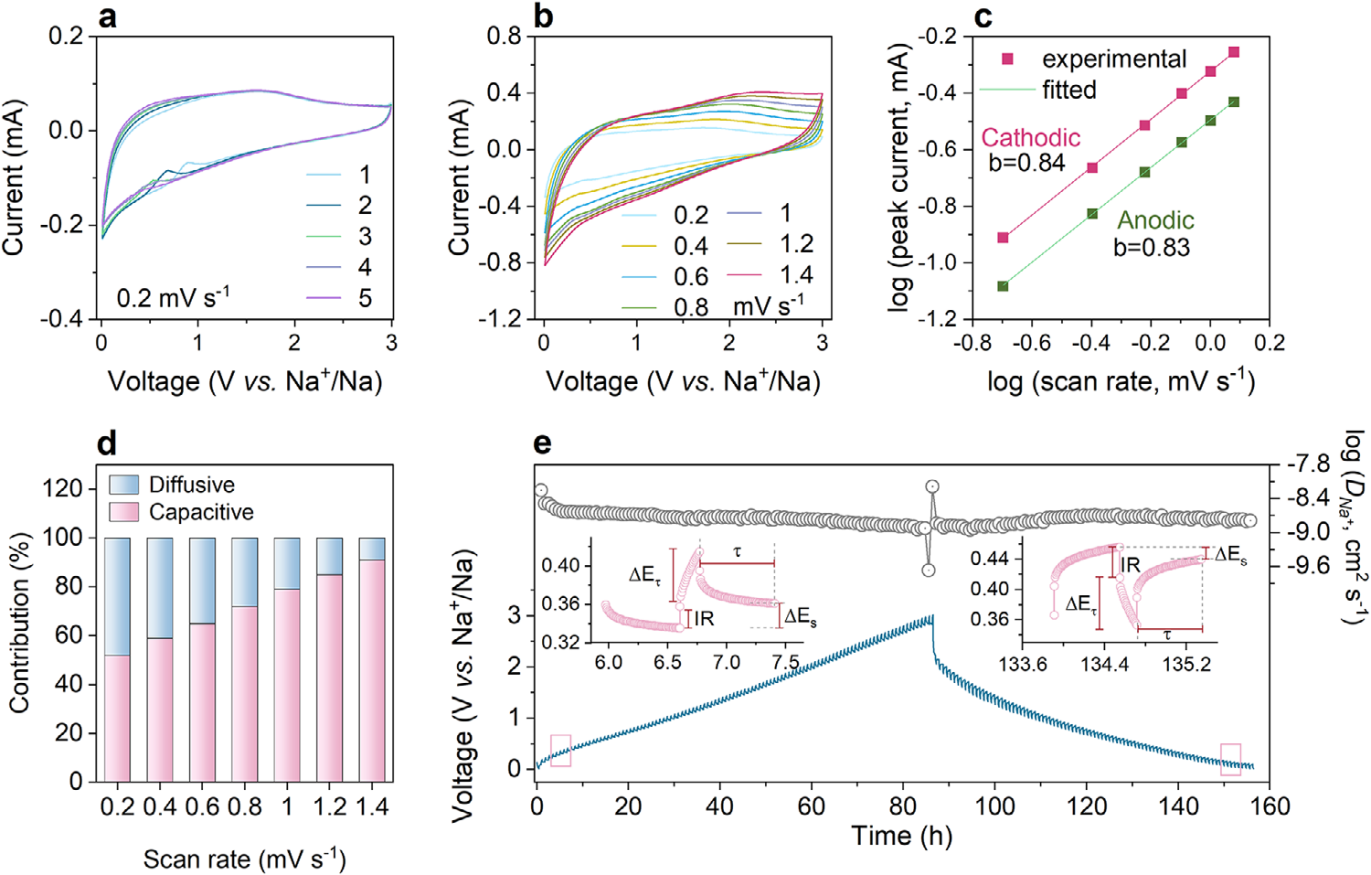
Kinetic studies of CVO*450* half‐cell. (a) CV curves of the initial five cycles at 0.2 mV s^−1^. (b) CV curves at different sweep rates (0.2 mV s^−1^ to 1.4 mV s^−1^), (c) linear plot of log(peak current) vs. log(scan rate). (d) Capacitive and diffusive contributions. (e) GITT plot at 0.1 A g^−1^ and corresponding diffusion coefficients (D_Na_^+^), with the inset showing parameters used for calculations.

Despite the high‐speed, surface‐dominated kinetics of the CVO*450* anode, long‐term cycling requires profound interfacial stability. The substantial growth of the SEI was dynamically tracked using stepwise operando EIS (Figure S9). During initial discharge, the charge‐transfer resistance (R*_CT_*) drops significantly, indicating electrochemical activation of the 2D diffusion channels [49, 50]. Subsequently, the emergence of SEI resistance (R*_SEI_*) below 1.0 V indicates the formation of the protective interphase necessary for stable, reversible capacitor performance. The EIS spectra were fitted by Randles’ circuit given in Figure S10, and corresponding complete details of fitted components are further highlighted in Table S3 and Note S3. To visually and chemically characterize this anode interphase, *ex situ* HRTEM was performed on the fully charged electrode, revealing a uniform, ∼18 nm thick SEI layer natively formed on the CVO micro‐rods (Figure 4a). The corresponding *i*‐FFT is displayed in the purple box [51]. Crucially, lattice fringe analysis identifies distinct (200) planes with an interplanar spacing of 0.23 nm, corresponding to highly crystalline, inorganic NaF. Driven by the decomposition of the NaPF_6_ salt and the FEC electrolyte additive, the formation of this highly inorganic, mechanically robust NaF‐rich SEI effectively passivates the active surface. It structurally isolates the “quasi‐zero‐strain” CVO framework from continuous parasitic electrolyte consumption, securing the necessary interfacial integrity for high‐rate, ultra‐long‐lifespan SICs. Additionally, the surface of CVO can be confirmed by the characteristic (005) planes and the corresponding *i*‐FFT pattern, representing an interplanar spacing of 0.21 nm in the yellow box. The dark‐field TEM image and EDS maps of Ca, V, O, C, F, P, and Na (Figure 4b) again confirm the uniform formation of the SEI layer over the surface of the CVO microrods.

**FIGURE 4:**
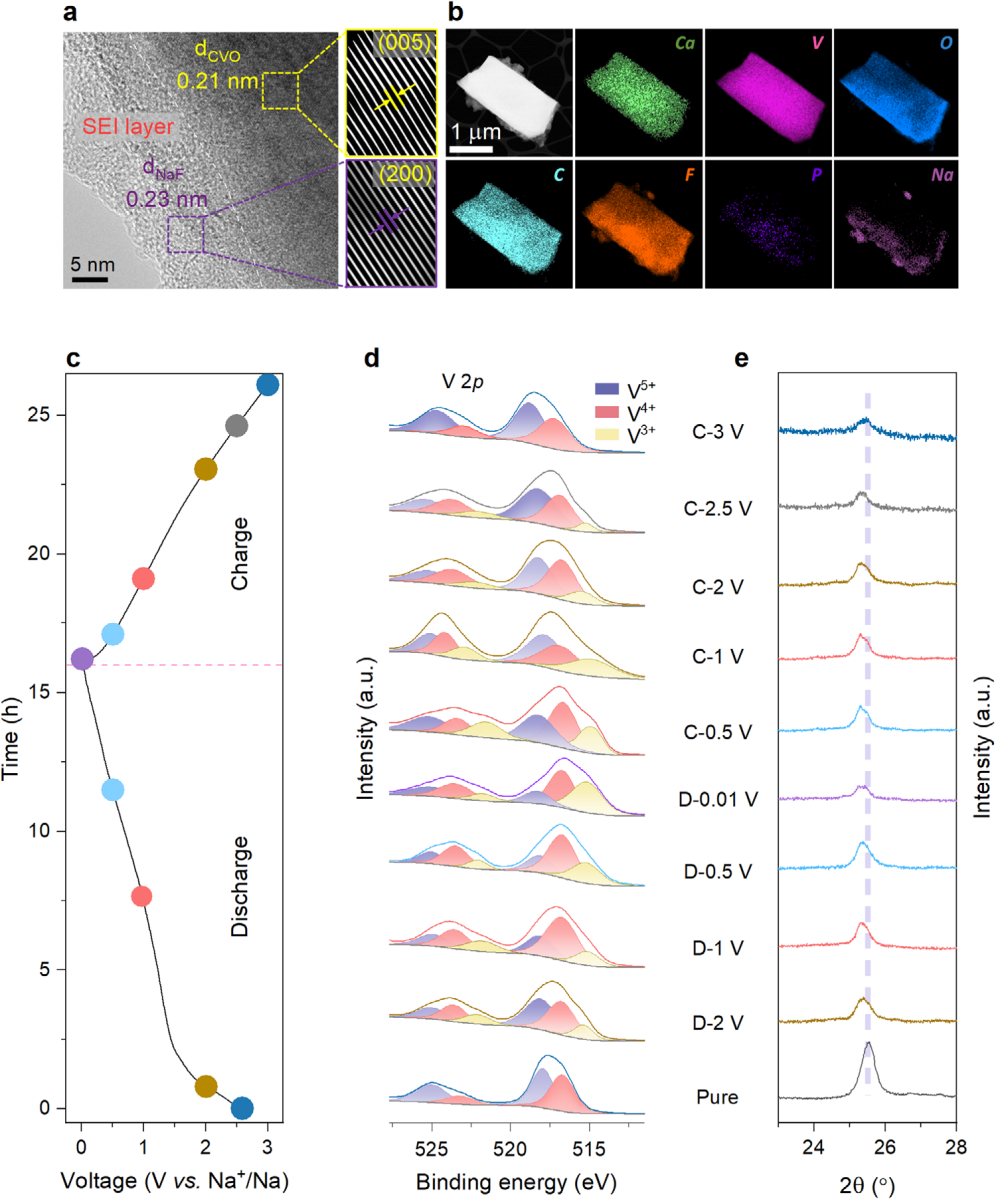
*Ex situ* investigations. (a) *Ex situ* HRTEM at a completely charged state of the cell exhibiting the planes corresponding to CVO and NaF. (b) EDS mapping displaying the distribution of Ca, V, O, C, F, P, and Na elements corresponding to the CVO and SEI layer at its edges. (c) The voltage vs. time profile shows the different points selected for measurement. *Ex situ* (d) XPS and (e) PXRD at different charging and discharging states.

To demonstrate the structural origin of this exceptional kinetic stability, the bulk‐phase evolution and redox activity were monitored using *ex situ* XPS and PXRD. First, *ex situ* XPS and PXRD were performed at different charge‐discharge states of the second cycle (Figure 4c–e). The XPS results show that during discharge, when Na^+^ ions intercalate into the anode, the oxidation state of V decreases, and a peak corresponding to V^3+^ becomes visible. This shows that, to maintain charge neutrality, vanadium is reduced (V^4+^ ↔ V^3+^) [34]. Moreover, the peak becomes significantly more intense when the cell is discharged to 0.01 V (Figure 4d). This peak further disappears when the CVO is charged to 3 V, exhibiting the reversibility and stability of our material. Simultaneously, there is a reduction from the 5+ to 4+ oxidation state (V^5+^ ↔ V^4+^) that is reflected by the corresponding peaks in the spectra [52]. The presence of mixed oxidation states is responsible for CVO*450*’s high specific capacity. Crucially, the oxidation state of the calcium remains entirely unchanged throughout deep sodiation and desodiation, validating its inert, structural‐supporting role (Figure S11). The defining proof of the “spectator effect” is observed in the *ex situ* PXRD evolution. Upon discharging to 0.01 V, the (110) peak at 25.6° shifts only marginally to lower angles, reflecting a highly restricted lattice expansion (Figure 4e). Overall, the framework demonstrates exceptional structural stability by rigidity, maintaining its interplanar spacing, which is 0.347 nm for pure and remains strictly preserved at 0.350 nm even at a deep discharge of 0.01 V. Rather than undergoing destructive metastable phase transitions, a fatal flaw in unpillared hydrated vanadates, the Ca^2+^ pillars effectively pin the edge‐shared VO_6_ polyhedra. Upon recharging to 3.0 V, the crystal structure precisely recovers its original *d*‐spacing. In addition, *ex situ* HRTEM performed after 600 cycles (Figure S12a,b) at a fully charged state shows retention of a 0.35 nm lattice spacing, further highlighting the exceptional long‐term structural robustness of the CVO framework. This highly restricted, reversible lattice breathing explicitly confirms a true “quasi‐zero‐strain” solid‐solution mechanism that perfectly accommodates the ultrafast pseudocapacitive flux without inducing mechanical fatigue. This remarkable retention of the pristine (003) interplanar spacing after 600 cycles provides direct evidence that the bulk crystal avoids mechanical strain.

To validate the “quasi‐zero‐strain” and spectator effect of Ca^2+^ ions in the CVO framework during the intercalation of Na^+^ ions, density functional theory (DFT) calculations were performed to evaluate bond lengths, integrated crystal orbital Hamilton populations (ICOHP), Bader charges, projected density of states (PDOS), and charge‐density differences. A multiscale computational workflow combining MACE‐based structural prescreening with final DFT optimization was employed to identify energetically stable pure and Na‐intercalated CVO configurations (all structures and corresponding energies are tabulated in Tables S4 and S5). Details of the bulk structural model construction, machine‐learning prescreening, and DFT calculations are provided in Note S4. The optimized pure and 1Na‐intercalated CVO structures are shown in Figure 5a, while 2Na‐ and 3Na‐intercalated configurations are given in Figure S13a.

**FIGURE 5:**
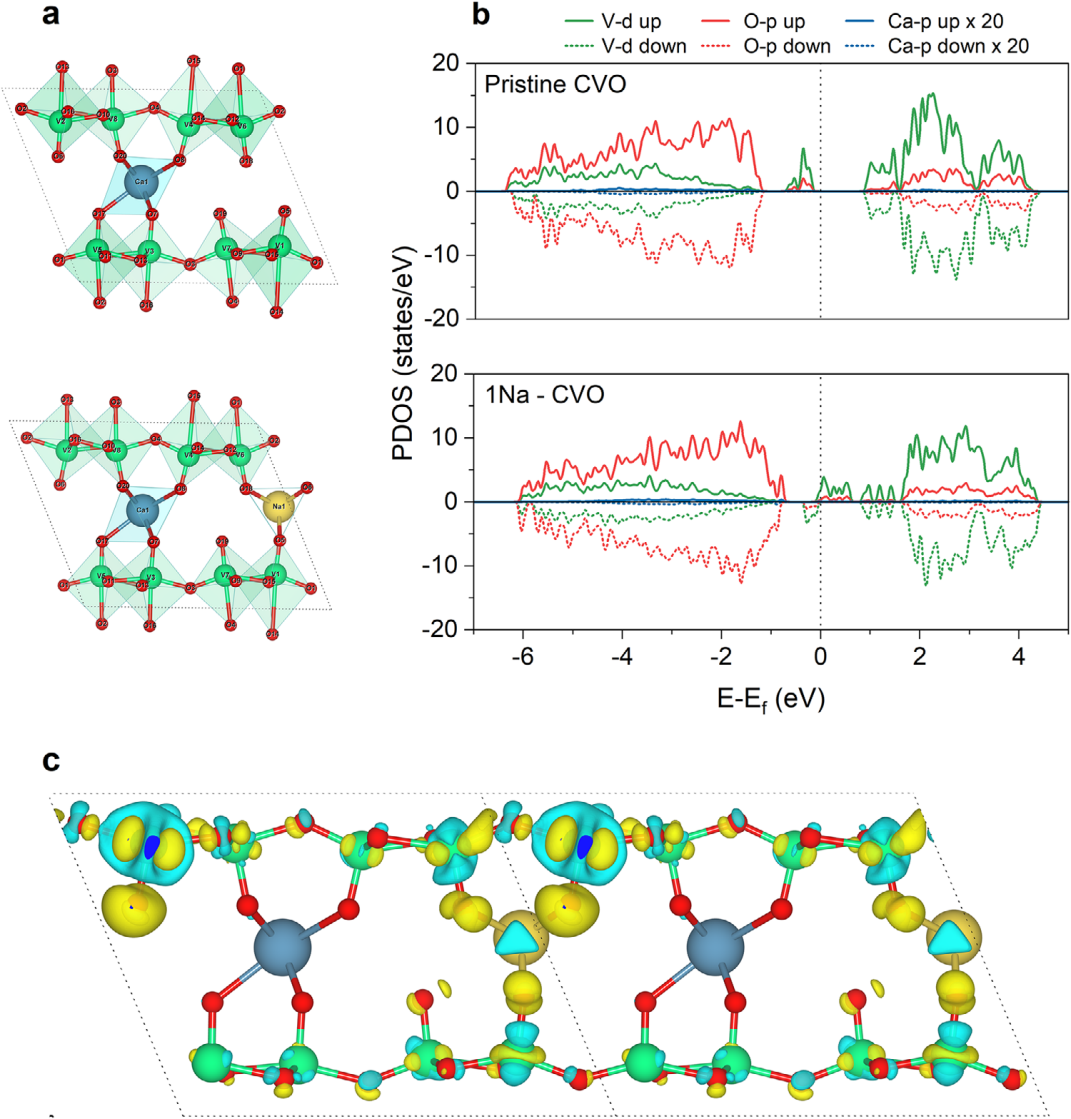
Theoretical analysis to identify the spectator effect of Ca^2^^+^ ions in CVO. (a) Optimized crystal structures of pure and Na‐intercalated CaV_8_O_20_. (b) Spin‐polarized projected density of states (PDOS) of pure and Na‐intercalated CaV_8_O_20_, highlighting the contributions of V 3*d*, O 2*p*, and Ca 3*p* orbitals. (c) Charge density difference upon Na intercalation, where yellow and cyan colors represent electron accumulation and depletion, respectively, indicating charge transfer from Na to the V─O framework.

Na^+^ insertion is expected to introduce electrostatic repulsion and promote lattice expansion in oxide frameworks. However, our optimized CVO structure exhibits only a minor volume change upon sodiation. The calculated unit cell volume increases from 381.784 Å^3^ for pure CVO to 386.022 Å^3^, 384.935 Å^3^, and 384.881 Å^3^ for the 1Na‐, 2Na‐, and 3Na‐intercalated structures, representing the volume expansions of only +1.11%, +0.83%, and +0.81%, respectively, as summarized in Table S6. Notably, the volume response is non‐monotonic as Na content increases, indicating that Na insertion is accommodated by local structural rearrangement rather than by uniform lattice expansion. This volume response is substantially smaller than previously reported for Na–intercalated V_2_O_5_, where a volume expansion of approximately 17% has been observed during sodiation [53].

To elucidate the origin of this minimal volume expansion, the evolution of Ca─O and V─O bonding was analyzed using DFT‐optimized bond lengths and ICOHP values (detailed Ca─O and V─O analyses are provided in Notes S5 and S6 and Tables S6 and S7, respectively). The ICOHP analysis reveals weak Ca─O interactions, as reflected in their low ICOHP values, confirming their predominantly ionic character. Upon Na insertion, the Ca─O coordination environment undergoes local elongation or contraction depending on the nearby Na arrangement, indicating that the weak Ca─O network provides structural flexibility for accommodating inserted Na^+^ ions. Unlike the weak Ca─O interactions, the V─O bonds show considerably larger −ICOHP values, confirming that the distorted VO_6_ polyhedra form the primary structural backbone of CaV_8_O_20_. During sodiation, selected V─O bonds undergo local elongation accompanied by reduced −ICOHP values, indicating selective weakening of specific V─O bonds while preserving the overall structural framework of CaV_8_O20.

The electronic structure further supports this bonding picture. The PDOS reveals negligible Ca‐projected contribution near the Fermi level (Figure 5b), indicating that Ca acts mainly as an electrochemically inactive structural pillar rather than a redox‐active center. Unlike Ca, the V 3*d* and O 2*p* states dominate the band‐edge region and exhibit strong hybridization, consistent with the higher V─O ─ICOHP values. Upon Na insertion, the occupied V 3*d* and O 2*p* hybridized states redistribute and partially shift toward lower energies below the Fermi level with increasing Na content (Figure 5b; Figure S13b), indicating Na‐to‐V‐O framework charge transfer and supporting partial reduction of V centers observed in *ex situ* XPS. This is consistent with the Bader charge analysis, where the inserted Na atoms undergo partial electron depletion, while the donated electron density is mainly accommodated within the V─O framework (Note S7and Table S8). In comparison, the Ca center shows only minor charge variation, further confirming its redox‐inactive role during sodiation.

The charge‐density‐difference plots further visualize this redistribution. In Figure 5c, electron depletion is observed around the inserted Na atoms, whereas electron accumulation is mainly localized around the neighboring V─O framework. Around Ca, no pronounced charge accumulation or depletion is observed, consistent with weak ionic Ca─O interactions, negligible Ca‐projected states near the Fermi level, and nearly unchanged Bader electron populations. Therefore, the charge‐density‐difference analysis supports the ICOHP, PDOS, and Bader results, confirming that the electrostatic perturbation introduced by Na^+^ insertion is locally compensated by the V─O framework rather than generating large‐scale volume expansion.

Overall, the minimal volume expansion of CVO during sodiation originates from a cooperative structural and electronic compensation mechanism. The weak ionic Ca─O coordination network provides local structural flexibility, while the stronger V─O framework undergoes selective bond weakening and accepts electron density from intercalated Na. This coupled local bond rearrangement and charge redistribution effectively accommodates Na^+^ intercalation and suppresses global expansion of the CVO lattice, thereby providing an atomistic origin for the experimentally observed minimal‐strain behavior.

### 2.3 Interfacial Stability via Scalable Chemical Pre‐Sodiation

To compensate for the initial active Na loss during SEI formation, a scalable chemical pre‐sodiation strategy was employed using a Na‐benzophenone (Na‐BPh) complex dissolved in diglyme (Dgy) ether. The underlying thermodynamic driving force for this rapid pre‐sodiation relies on the highly reducing nature of the BPh radical anions (Scheme 1). The strong reducing property of Na metal results in the formation of BPh^−^ radicals. These radicals turned the BPh/Dgy solution dark blue within 3 min, forming a Na‐BPh/Dgy solution (Figure 6a,b). The solution remained stable for 6 months, demonstrating the durability of the process (Figure 6c). In Figure 6d, Electron paramagnetic resonance (EPR) spectra containing the sharp set of peaks (g = 2.0068) confirm the formation of Na‐BPh ketyl radicals [54]. This demonstrates electron transfer from Na metal to BPh, forming the Na‐BPh species. The strong reducing properties of Na‐BPh help in the pre‐sodiation of CVO to form Na_x_CVO (explained by the schematic in Figure S14). According to frontier molecular orbital theory, the highest occupied molecular orbital (HOMO) of the Na‐BPh complex aligns favorably with the lowest unoccupied molecular orbital (LUMO) of the CVO electrode, facilitating spontaneous electron transfer from the BPh^−^ radical to the CVO framework with concurrent Na^+^ insertion [24].

**SCHEME 1:**
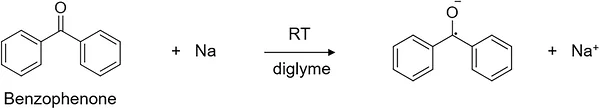
The reaction mechanism for the formation of Na‐BPh reducing agent.

**FIGURE 6:**
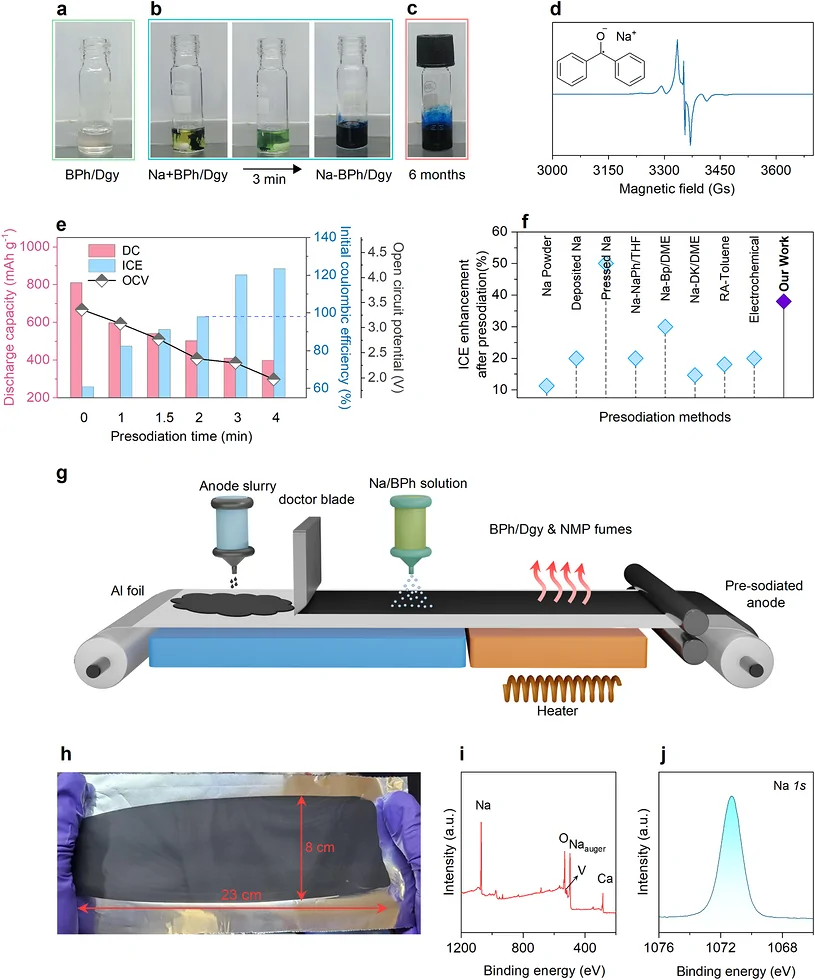
Pre‐sodiation optimizations. Optical photographs of (a) BPh/Dgy, (b) Na dissolved BPh/Dgy reagent at different intervals, and (c) stable blue‐colored Na‐BPh/Dgy solution after 6 months, confirming long‐term chemical stability. (d) EPR spectra verifying the formation of BPh radicals in the Na‐BPh/Dgy solution (inset: molecular structure of the BPh radical). (e) Comparative bar graph of ICEs, DCs, and OCV of half cells packed with CVO anode pre‐sodiated for different durations. (f) Graph comparing the ICE retention achieved by different pre‐sodiation methods [24, 55, 56, 57, 58, 59, 60, 61, 62]. (g) Schematic illustration of laboratory‐scale roll‐to‐roll approach and corresponding (h) coated foil. The incorporation of Na^+^ ions within the CVO framework during pre‐sodiation is observed by XPS (i) survey spectra and (j) high‐resolution Na 1*s* spectra after 5 min of surface etching.

To control the degree of pre‐sodiation, CVO*450* electrodes were soaked in Na‐BPh/Dgy solution for different intervals (0 min, 1 min, 1.5 min, 2 min, 3 min, and 4 min). For the complete pre‐sodiation process, refer to the experimental section. Figure 6e compares the initial discharge capacities (DCs), open circuit voltages (OCVs), and corresponding ICEs of half‐cells pre‐sodiated for different intervals. During soaking, the number of Na^+^ ions intercalated into CVO increases. It can be understood that the insertion of Na^+^ at the anode decreases the redox potential of the half‐cell, and the OCV of the half‐cell eventually falls. The OCV for non‐sodiated cells was 3.36 V, which dropped to 1.97 V after 4 min of soaking. This helps compensate for the initial loss of active ions during SEI formation. The bar graph shows that after soaking the anode electrode for 2 min, sufficient Na^+^ ions are inserted to achieve an ICE close to 100% (for more details, see Figure S15 and Table S9). The ICE increases from 60.86% to 98.03% after 2 min of pre‐sodiation. This ICE enhancement (∼38%) is compared with other previously reported pre‐sodiation approaches (Figure 6f), including direct contact [55, 56, 63], electrochemical [57], and other chemical pre‐sodiation methods [58, 59, 60, 61]. This showcases the remarkable effectiveness and superiority of our method for SICs. It is noteworthy that this approach is faster than other pre‐sodiation methods, such as electrochemical (∼30 h) [25], direct contact (∼5 h) [20, 21] and sacrificial salts (∼10 h) [24].

Figure 6g schematically depicts a roll‐to‐roll assembly process in which the CVO slurry is continuously coated onto an Al foil substrate, followed by in‐line pre‐sodiation via controlled spraying of the Na–BPh/Dgy solution (a complete explanation is given in the Experimental Section). Highlighting its industrial compatibility, this gas‐free chemical strategy was successfully translated into a continuous laboratory‐scale roll‐to‐roll manufacturing process. The strategy was utilized to fabricate large‐area (23 × 8 cm) pre‐sodiated electrodes with high uniformity (Figure 6h). Further, *ex situ* XPS confirms the deep incorporation of Na^+^ ions in the CVO structure. The pre‐sodiated CVO electrode was first etched with Ar^+^ ions for 5 min (∼20 nm) to remove surface contaminants. The survey spectra and high‐resolution Na 1*s* spectra confirm the presence of ions in the CVO framework (Figure 6i,j).

### 2.4 Full Cell Sodium Ion Capacitor (SIC) Performance

This pre‐sodiated structurally robust CVO*450* anode was used in a full‐cell SIC with a carbon‐based cathode (the assembly shown in Figure 7a). To prevent a kinetic mismatch and thermodynamically balance the high specific capacity of the pre‐sodiated CVO*450* anode, a standard AC cathode is insufficient. Therefore, we engineered an Activated Carbon/Soft Carbon (AC/SC) composite cathode (details of electrode preparation are given in the Experimental section. Alongside, the electrochemical performance of the AC/SC cathode was also investigated in a half‐cell configuration to identify its capacitive and cathodic properties at higher voltages (Figure S16). The GCD plots in Figure 7b show the overlapping of the GCD plots of anodic CVO*450* and cathodic carbon materials. In this hybrid device, the kinetic mismatch typical of SICs is effectively bridged: the porous carbon cathode operates via rapid, non‐faradaic anion adsorption/desorption, while the pre‐sodiated CVO*450* anode, supported by its Ca^2+^ pillared enlarged interlayers and the robust NaF‐rich protective SEI, sustains ultrafast Na^+^ solid‐solution intercalation without structural degradation. Figure 7c displays the GCD curves of SIC at different current rates over a voltage window of 0.01 V to 3.6 V. Significant performance at higher rates is again attributed to the pillared structure. Furthermore, CV at 2 mV s^−1^ indicates a semi‐rectangular shape that exhibits a redox‐peak‐free profile, thereby confirming the deep pseudocapacitive charge‐storage kinetics behavior of the SIC (Figure 7d). In addition, Figure S17 shows the CV curves of SIC at different scan rates ranging from 5 to  50 mV s^−1^.

**FIGURE 7:**
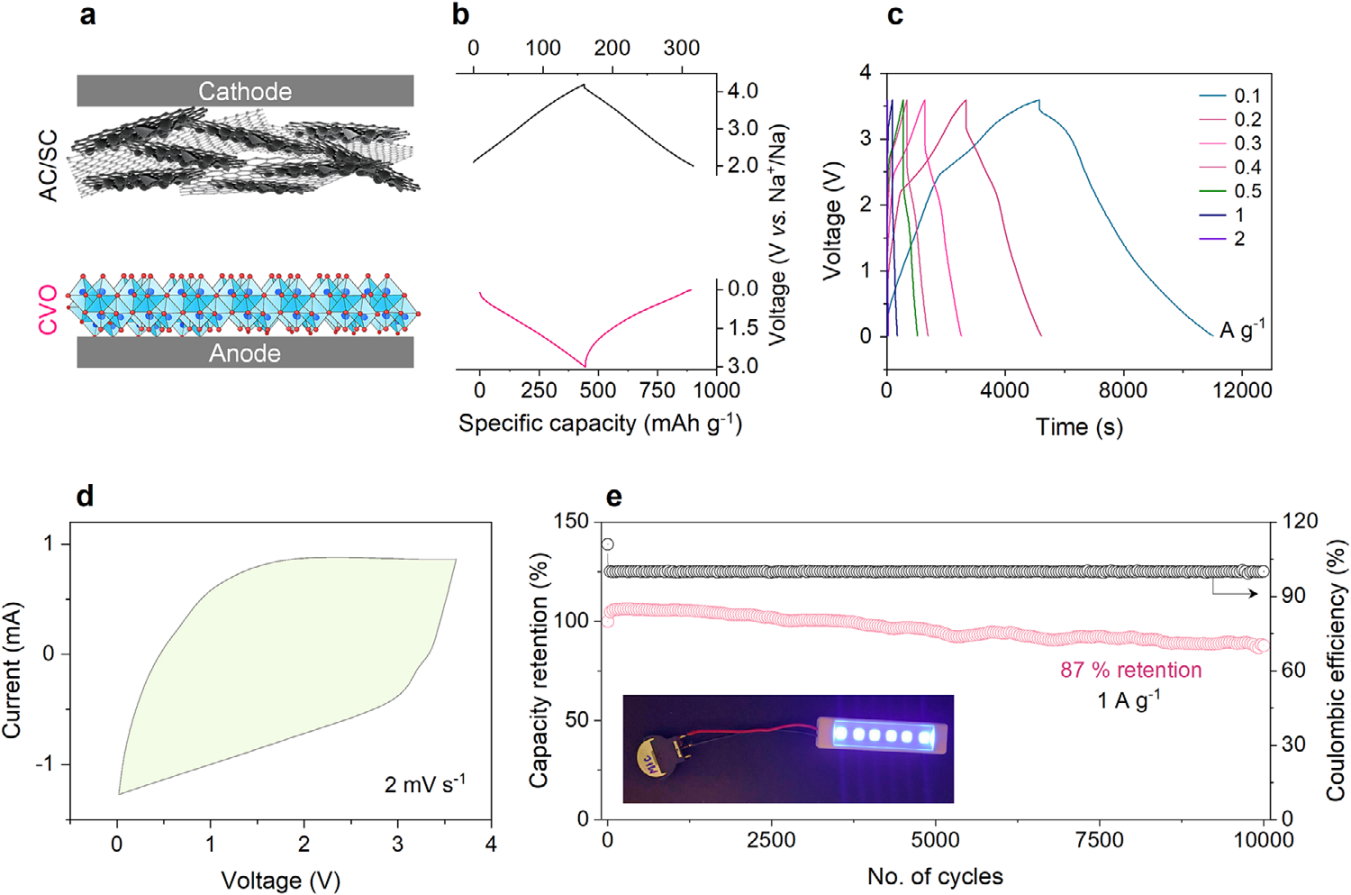
Full cell investigations. (a) Full cell SIC assembly. (b) Overlapped GCD curves of CVO and AC/SC cathode. (c) GCD curves of SIC at different current rates and (d) CV curve at 2 mV s^−1^. (e) Cycle stability test at 1 A g^−1^ (inset showing the cell could glow under a UV light panel).

The fabricated SIC exhibits remarkable electrochemical metrics, delivering material‐level specific energy of 176 Wh kg^−1^ at a specific power of 380 W kg^−1^, based on the active mass on both the electrodes (for calculation details, see Equations S3–S5 in Note S2). The results outclass several previous reports, including VN@C//AHPC (103 Wh kg^−1^@115 W kg^−1^) [63], TiO_2_/C‐HPD_3_ (147.9 Wh kg^−1^@360 W kg^−1^) [64], and SbWSe/C/NF (120.49 Wh kg^−1^@ 380 W kg^−1^) [65]. To benchmark the electrochemical superiority of the assembled device, a Ragone plot (Figure S18) was constructed. The plot demonstrates the synergy between the pre‐sodiated CVO anode and the AC/SC composite cathode, enabling the device to outperform state‐of‐the‐art SICs. To rigorously evaluate its practicability, the device‐level SE was calculated by also incorporating the masses of inactive components (current collectors, binders, separators, and electrolyte), yielding an impressive practical energy density of 18.7 Wh kg^−1^ (detailed calculations are provided in Note S8). Furthermore, the fully optimized SIC exhibits an ultra‐long lifespan, retaining 87% of its initial capacity after 10,000 cycles at a high current density of 1 A g^−1^ (Figure 7e). This exceptional durability (compared in Table S10), capable of continuously powering a UV LED panel (inset of Figure 7e) without notable voltage decay, validates that combining “quasi‐zero‐strain” structural pre‐intercalation with a scalable, gas‐free chemical pre‐sodiation protocol provides a definitive and competitive pathway for developing state‐of‐the‐art sodium‐ion hybrid capacitors.

## 3 Conclusions

This study establishes a synergistic design paradigm for next‐generation SICs by coupling a structurally engineered “quasi‐zero‐strain” calcium vanadate (CaV_8_O_20_) anode with an ultrafast, laboratory‐scale chemical pre‐sodiation protocol. Mechanistically, stepwise operando EIS and *ex situ* structural analyses reveal that Ca^2+^ entities exert a robust ‘spectator effect’. Acting as electrochemically inactive pillars, they prevent framework collapse and enable highly reversible Na^+^ solid‐solution kinetics, ensuring 90% capacity retention over 600 cycles in half‐cells. To overcome the fatal flaw of initial active Na loss, an ultrafast roll‐to‐roll chemical pre‐sodiation was successfully implemented. Driven by HOMO/LUMO energy alignments, the Na‐BPh/Dgy solution enables spontaneous electron transfer, achieving nearly 100% ICE within just 2 min of soaking, demonstrating a time‐efficient approach. This approach exclusively uses pure Na metal and is gas‐free, thereby maximizing pre‐sodiation efficiency. The industrial compatibility of this method was represented by the laboratory‐scale roll‐to‐roll pre‐sodiation of the CVO anode. Consequently, the fully assembled pre‐sodiated SIC overcomes traditional kinetic imbalances, delivering an exceptional specific energy of 176 Wh kg^−1^ at a high specific power of 380 W kg^−1^. The robust structural and interfacial engineering endows the device with an ultra‐long lifespan, retaining 87% of its initial capacity after 10,000 cycles at 1 A g^−1^. This work not only provides a scalable, continuous manufacturing pathway for high‐performance SICs but also establishes a foundational pre‐sodiation platform. Furthermore, utilizing operando characterization to probe the dynamic evolution of these ether‐derived, NaF‐rich interphases under extreme temperatures and ultra‐fast charging conditions will be critical to fully unlocking the commercial potential of advanced sodium‐ion technologies.

## 4 Experimental Section

### 4.1 Materials

Vanadium(V) oxide (V_2_O_5_, > 98%), polyvinylidene difluoride (PVDF) binder, Na metal, diglyme (diethylene glycol dimethyl ether), and n‐methyl 2‐pyrrolidone (NMP, 99.5%) were supplied by Sigma‐Aldrich. Calcium chloride (CaCl_2_ fused), benzophenone, and hydrogen peroxide (H_2_O_2_) were obtained from SD‐Fine suppliers. For electrolyte preparation, sodium hexafluorophosphate (NaPF_6_, 98%), ethylene carbonate (EC, 98%), diethyl carbonate (DEC, 99%), and fluoroethylene carbonate (FEC, > 99%) were also purchased from Sigma‐Aldrich. Activated carbon (AC) was purchased from Global Nanotech. Soft carbon (SC) was synthesized by our previously reported method [66]. Deionized water (DI) was used from a laboratory‐installed Elga water purifier (18 MΩ, purity > 99%).

### 4.2 Synthesis of CVO Nanobelts

To prepare the CVO microbelts, 2 mmol V_2_O_5_ was mixed in 30 mL of DI water. After continuous stirring for 15 min at 80°C, 5 mL of H_2_O_2_ was added dropwise, and the solution was stirred for an additional 0.5 h. Further, 10 mmol of CaCl_2_ was added, and the mixture was stirred for an additional 1 h to obtain a homogeneous, dark orange solution. During the process, fumes were observed, signifying an exothermic reaction. The homogenized mixture was transferred to a 50 mL Teflon‐lined autoclave and heated to 220°C for 48 h. After the reaction cooled naturally, the dark orange‐coloured powder was obtained by washing it with DI water and methanol several times to remove any residual material. Finally, the obtained sample was dried overnight at 60°C. Further, the samples were annealed in an inert atmosphere at 350°C, 450°C, and 550°C for 2 h, designated CVO*350*, CVO*450*, and CVO*550*, respectively.

### 4.3 Preparation of Na‐BPh/Dgy Solution

To prepare the Na–BPh/Dgy solution, 1 mmol of benzophenone was added to 1 mL of diglyme. It was stirred for 10 min to achieve a clear solution. Further, 1 mmol of Na metal was added to the solution, which was stirred for 2 h to obtain a dark blue solution. The complete process was performed in the Ar‐filled glove box (H_2_O < 0.1 ppm, O_2_< 0.1 ppm).

### 4.4 Preparation of CVO Electrode

First, the slurry for the anode was prepared by dissolving CVO samples, carbon black, and PVDF in an 8:1:1 ratio (by weight), respectively, in NMP solvent. To achieve uniform dispersion of the active material, the slurry was mixed in a slurry maker for ∼15 min and then coated onto Al foil using a tape‐casting coater. After vacuum‐drying the coated foil at 120°C, electrodes with a 15 mm diameter were punched using a disc cutter. The cathode electrodes were prepared using the same process, with AC, SC, and PVDF (6:3:1 wt%) as slurry precursors.

### 4.5 Pre‐Sodiation of CVO Electrode

For a full‐cell SIC, the CVO*450* electrodes were first pre‐sodiated by immersing them in Na–BPh/Dgy solution for different time intervals (1–4 min). Subsequently, the electrodes were thoroughly rinsed with diglyme to eliminate residual blue solution and remove any excess pre‐sodiation reagent from the surface.

The chemical pre‐sodiation approach was scaled up to large‐scale production via a roll‐to‐roll process. During the coating process, a roll of Al foil was passed over the rollers, and simultaneously, the CVO material slurry was dispensed onto the foil. Meanwhile, the doctor blade smoothed the slurry, followed by the spraying of the Na‐BPh/Dgy solution. Lastly, the foil was conveyed through a heating zone at 100°C to evaporate residual solvents and complete electrode formation.

### 4.6 Electrochemical Testing

For the electrolyte, 1 m NaPF_6_ was dissolved in EC:DEC (1:1 by volume) and 5 vol% FEC solvent. Whatman filter paper was used as a separator in both half‐ and full‐cell devices. The half‐cells were packed using CVO and carbon electrodes against Na metal foil to analyze their respective anodic and cathodic characteristics. These coin cells (CR2032) were packed using an e‐crimper (19.6 MPa pressure) in the glove box (O_2_ < 2 ppm; H_2_O < 0.1 ppm). Because of the water content in the CVO*p* and CVO*350* samples, only CVO*450* and CVO*550* were included for electrochemical measurements.

Furthermore, the full cell was packed using a pre‐sodiated CVO*450* anode and an AC/SC cathode with a 1:3 mass ratio. The mass ratios were determined by calculating the corresponding capacity ratios based on the half‐cell performances of the anode and cathode. The GCD and long‐term cycling performance were obtained using the Neware Battery Test System at room temperature. The voltage windows of 0.01–3 V and 0.01–3.6 V were chosen for half‐ and full‐cell applications, respectively. CV and EIS for both cells were acquired using a Biologic VMP3 potentiostat at room temperature. For the stepwise operando EIS study (frequency range: 10 mHz to 100 kHz; amplitude: 5 mV), the cell was charged/discharged to the required voltage limits, followed by a 1‐h rest to allow the cell to reach equilibrium. For each EIS plot, EC Lab11.60 software was used to obtain the corresponding Randles’ circuit fit.

### 4.7 Physical Characterization

The crystal structure of the CVO is characterized by Powder X‐ray diffraction (PXRD) equipped with Cu Kα radiation (λ = 1.5406 Å) on a Malvern Panalytical Empyrean instrument. The X‐ray photoelectron spectroscopy (XPS; Al K*α* radiation) instrument (Thermo Fisher Scientific, Model Nexa) was used to determine the chemical composition of all samples. The morphology of the samples was characterized by FESEM (JEOL, JSM‐7900F) at 8 keV. The HRTEM images and SAED patterns were recorded using JEOL JEM‐ARM 200F NEOARM at 200 keV. Gatan Digital Micrograph software was used to analyze the HRTEM images and SAED patterns. An image was used to draw the *i*‐FFT patterns from HRTEM images. Further, elemental mapping was performed using an EDS detector on the HRTEM instrument. For *ex situ* HRTEM, the cell was decrimped in the glove box after 2 cycles, and the electrode was rinsed 3–4 times with DEC to remove electrolytes and other impurities. Further, the CVO electrode material is dispersed in DEC solvent and drop‐cast onto a lacey carbon TEM grid. The grid was dried overnight and transferred to an inert gas‐filled box for further investigation. The TGA was conducted on a TA Instruments SDT 650. EPR spectra were recorded at 100 K on a Bruker Biospin EMXmicro (A300) spectrometer. Surface area and pore size measurements were performed using an Autosorb‐iQ (Quantachrome) surface area analyzer at a temperature of 77 K. A Nicolet IS50 Thermo Scientific instrument was used for Fourier transform infrared (FTIR) measurements. To perform *ex situ* XPS, HRTEM, and PXRD measurements, the half‐cells were opened at specific charged/discharged voltages and then rinsed with DEC 3–4 times to remove surface impurities.

## Author Contributions

**Neetu Bansal**: methodology, software, data curation, investigation, validation, formal analysis, visualization, writing – original draft, writing – review and editing. **Anwar Hussain**: methodology, data curation, formal analysis, writing – original draft. **Radhey Shyam Yadav**: software, data curation, formal analysis, Writing – original draft, Writing – review and editing, visualization, investigation. **Prakash Kumar Pathak**: methodology, validation, visualization, writing – original draft, formal analysis. **Minjoong Kim**: methodology, visualization, formal analysis, data curation. **Tobias Mattisson**: data curation, software, validation. **Heejoon Ahn**: project administration, supervision, writing – original draft, writing – review and editing. **Rahul R. Salunkhe**: conceptualization, project administration, resources, supervision, writing – original draft, writing – review and editing, funding acquisition, investigation, methodology.

## Funding

R. R. S. is grateful for the funding support received from the DST through the following grants: DST‐FIST grant SR/FST/PS‐I/2023/234 and DST‐NEST grant DST/CEST/NEST/2024/241. We also appreciate the funding from DRDO, specifically NRB/4003/PG/MAT/556, as well as the manpower support provided by the PMRF. Additionally, we would like to thank IIT Jammu for offering the PRAISE research grant.

## Conflicts of Interest

The authors declare no conflict of interest.

## Supporting information




**Supporting File**: advs77176‐sup‐0001‐SuppMat.docx.

## Data Availability

The data that support the findings of this study are available in the supplementary material of this article.
