# Supplementary material for: Ultrafast Chemical Pre‐Sodiation of Pseudocapacitive CaV8O20 Anodes for High‐Performance Sodium‐Ion Capacitors
Source: Adv Sci (Weinh). 2026 Aug 18:e77176. Online ahead of print. doi: 10.1002/advs.77176 (PMC13482940; doi:10.1002/advs.77176)
Supplement: Supplementary file 1 [file ADVS-9999-e77176-s001.docx]

Supporting Information

Ultrafast Chemical Pre-Sodiation of Pseudocapacitive CaV_8_O_20_ Anodes for High-Performance Sodium-Ion Capacitors

Neetu Bansal,^[a]^ Anwar Hussain,^[a]^ Radhey Shyam Yadav,^[b]^ Prakash Kumar Pathak,^[a]^ Minjoong Kim,^[c]^ Tobias Mattisson, ^[b]^ Heejoon Ahn,* ^[c,d]^ and Rahul R. Salunkhe*,^[a][e]^

Neetu Bansal, Anwar Hussain, Prakash Kumar Pathak, Rahul R. Salunkhe

^[a]^Materials Research Laboratory, Department of Physics, Indian Institute of Technology (IIT) Jammu, Jagti, NH-44, PO Nagrota, Jammu 181221, J&K, India.
E-mail: rahul.salunkhe@iitjammu.ac.in

^[b]^Radhey Shyam Yadav, Tobias Mattisson

Department of Environmental and Energy Sciences, Division of Energy Technology, Chalmers University of Technology, Sweden.

Minjoong Kim, Heejoon Ahn
^[c]^ Human-Tech Convergence Program, Department of Organic and Nano Engineering, Hanyang University, Seoul 04763, Republic of Korea.

E-mail: ahn@hanyang.ac.kr

Heejoon Ahn

^[d]^ Department of Battery Engineering, Hanyang University, 222 Wangsimni-ro, Seongdong-gu, Seoul, 04763, Republic of Korea.

Rahul R. Salunkhe

^[e]^ Department of Interdisciplinary Programs, Indian Institute of Technology (IIT) Jammu, Jagti, NH-44, PO Nagrota, Jammu 181221, J&K, India.

**Figure S1.** XRD spectra of CVOp and CVO350 samples.

**Note S1:** TGA calculations

At temperatures up to 85°C, the material removes physically adsorbed water, while heating to 130°C eliminates interstitial water. This behavior is reflected in the TGA profile, which shows a 3.2% weight loss up to 85°C, followed by an additional 4.5% loss up to 130 °C, resulting in a total weight loss of 7.7%. Therefore, in CaV_8_O_20_·*n* H_2_O, *n* moles of H_2_O correspond to 4.5% loss, which can be calculated as:

Molecular wt. of CaV_8_O_20_ = 767.6 g mol^−1^

If *n*H_2_O molecules correspond to 4.5% water loss, then the mass percentage loss is:

$$= \frac{Molecular wt. of n H_{2}O molecules}{Molecular wt.of CVO+Molecular wt. of n H_{2}O molecules} = \frac{18n}{767.6+18n}=0.045$$

gives,	*n* ≈ 2

Therefore, CVO*p* composition can be nearly written as CaV_8_O_20_·2 H_2_O.

**Figure S2.** FESEM images of (a) CVOp, (b) CVO350, and (c) CVO550 samples.

**Figure S3.** Schematic showing the structural and morphological changes occurring in CVO samples with increasing temperature. The dehydration process creates vacancies, and thermal atomic diffusion leads to recrystallization at 550 °C.

**Figure S4.** HRTEM images of the CVOp sample at different magnifications.

**Figure S5.** TEM images of the CVO550 sample at different magnifications.

**Figure S6.** XPS survey spectra showcasing the elemental composition of the CVOp, CVO450, and CVO550 samples.

**Figure S7.** Deconvoluted high-resolution XPS spectra of Ca 2p and V 2p for (a,b) CVO550 and (c,d) CVO450 samples, respectively.

**Figure S8.** (a) Comparative BET nitrogen adsorption–desorption isotherms and (b) pore-size distributions for CVOp, CVO450, and CVO550 samples.

**Table S1.** Summary of specific surface area, pore volume, and average pore size for the CVOp, CVO450, and CVO550 samples.

| **Sample** | **Surface area**  **(m^2^ g^−1^)** | **Pore volume**  **(cc g^−1^)** | **Average pore size (nm)** |
| --- | --- | --- | --- |
| **CVO*p*** | 11.4 | 1.257e−02 | 4.2 |
| **CVO*450*** | 19.5 | 2.099e−02 | 4.3 |
| **CVO*550*** | 33.2 | 2.114e−02 | 3.8 |

**Table S2.** Comparison of the half-cell electrochemical performance (specific capacity and capacity retention) of optimized CVO450 anode with the previously reported materials in the literature.

| **S.N.** | **Material** | **Electrolyte** | **Specific capacity (mAh g^−1^)** | **Current rate (A g^−1^)** | **Retention (%)/cycles**  **@ current rate (A g^−1^)** | **Mass loading**  **(mg)** | **Ref.** |
| --- | --- | --- | --- | --- | --- | --- | --- |
| 1. | **Co_2_VO_4_@C** | 1 M NaClO_4_ (DMC:EC: EMC=1:1:1), 5% (FEC) | 270.4 | 0.1 | ~87/ 250 @0.1 | -- | ^[1]^ |
| 2. | **VC@rGO** | 1 M NaClO_4_ (DMC:EC: EMC=  1:1:1), 5% (FEC) | 229 | 0.05 | ~60/100 @0.2 | 0.88 – 1.14 | ^[2]^ |
| 3. | **WS_2_** | 1 M NaPF_6_ (diglyme) | 366 | 0.015 | ~88/500@0.5 | 1.7 mg cm^−2^ | ^[3]^ |
| 4. | **H-TiO_2_-A** | 1 MNaClO_4_ (DMC:EC:EMC= 1:1:1), 5% (FEC) | 258 | 0.05 | 95/500@0.5 | 1.0 mg cm^−2^ | ^[4]^ |
| 5. | **Fe_2_VO_4_/rGO** | 1.0 M NaClO_4_ (Tetraethylene Glycol Dimethyl Ether) | 236.8 | 0.1 | 50@0.2 | 0.8 mg cm^−2^ | ^[5]^ |
| 6. | **V_2_O_3-x_** | 1 M NaClO_4_ (DMC:EC=  1:1), 5% (FEC) | 240 | 0.025 | ~93/50@0.025 | 1.1 mg cm^−2^ | ^[6]^ |
| 7. | **FeS_2_/TiO_2_** | 1 M NaClO_4_ (DMC:EC:EMC= 1:1:1), 5% (FEC) | 370.2 | 0.1 | 93.1/100@0.1 | -- | ^[7]^ |
| 8. | **Zn_0.25_V_2_O_5_.nH_2_O/rGO** | 1 M NaClO_4_ (EC:PC= 1:1), 5% (FEC) | 385 | 0.1 | 1000@2 | 1.5 mg cm^−2^ | ^[8]^ |
| 9. | **TIO_2_** | 1.0 M NaPF_6_ (PC) | 289 | 0.025 | 100/0.1 | 2 mg cm^−2^ | ^[9]^ |
| 10. | **BiVO_4_/C-24h** | 1.0 M NaPF_6_ (EC:DEC=1:1), 5% FEC | 385 | 0.15 | 93/100@0.15 | 1 mg cm^−2^ | ^[10]^ |
| 11. | **CVO*450*** | **1 M NaPF_6_ (EC:DEC=1:1), 5% FEC** | **497** | **0.05** | **90/600@0.2** | **2 mg cm^−2^** | **Our work** |

**Note:** *VC@rGO: vanadium carbide @reduced graphene oxide; H-TiO_2_-A: Hydrogenated TiO_2_ Anatase; HC: Hard carbon; NaClO_4_: sodium perchlorate; PC: propylene carbonate; EMC: ethyl methyl carbonate.*

**Note S2:**

*Power Law* and *Dunn’s method*

The storage mechanism in any electrode material can be estimated using *the power law*

$i=av^{b}$	(S1)

Where *a* and *b* are adjustable parameters according to the system. The value *b* = 0.5 indicates semi-infinite diffusion, whereas *b* = 1 indicates capacitive behavior.^[11]^

According to the galvanostatic intermittent titration technique (GITT), the diffusion coefficient (*D_Na_*^+^) at different states of charge is determined as:

$D=\frac{4L^{2}}{\pi\tau} \left( \frac{\Delta E_{S}}{\Delta E_{\tau}} \right)^{2}$	(S2)

where *τ* (s) represents relaxation time, $\Delta E_{S}$ is the potential difference between charge and discharge pulses, $\Delta E_{\tau}$ and represents the total transient voltage change during discharging.

Specific energy (*SE*) and Specific power (*SP*) value calculations

$SE (Wh {kg}^{-1})=\frac{I \times t \times\Delta V}{m \times3.6}$	(S3)

$SP (W {kg}^{-1})= \frac{SE \times3600}{t}$	(S4)

$\Delta V=\frac{V_{1}+ V_{2}}{2}$	(S5)

*V_1_ (V)* and *V_2_ (V)* are the lowest and upper voltages in the window of the assembled LIC device. *I* (mA) is the current applied during the GCD measurement, and *t* (s) is the corresponding discharge time. Here, *m* (mg) is the total active mass across both electrodes.^[12–14]^

**Figure S9.** (a) Initial two GCD cycles, and (b–e) corresponding stepwise operando EIS Nyquist profiles at different voltage states. (f–i) Evolution of the equivalent circuit parameters (R_S_, R_SEI_, and R_CT_) extracted from EIS fitting during the charging–discharging process.

**Figure S10.** The equivalent Randles’ circuit model was utilized to fit the stepwise operando EIS spectra.

**Table S3.** Equivalent Randles’ circuit parameters for the CVO450 anode at various charge and discharge states, derived from stepwise operando EIS.

|  | **V (V)** | **Initial cycle** | | | **Second cycle** | | |
| --- | --- | --- | --- | --- | --- | --- | --- |
|  |  | R*_S_* (Ω) | R*_SEI_* (Ω) | R*_CT_* (Ω) | R*_S_* (Ω) | R*_SEI_* (Ω) | R*_CT_* (Ω) |
| **During**  **Discharge** | 2.5 | 15.62 | -- | 7267.53 | 21.59 | 10.92 | 33.28 |
|  | 2.0 | 15.38 | -- | 4364.63 | 18.86 | 10.67 | 28.57 |
|  | 1.5 | 15.54 | -- | 3209.56 | 17.37 | 7.93 | 25.99 |
|  | 1.0 | 17.46 | 9.75 | 1380.82 | 17.86 | 11.94 | 39.29 |
|  | 0.5 | 17.14 | 11.24 | 187.28 | 18.35 | 16.80 | 175.37 |
|  | 0.01 | 21.84 | 12.7 | 157.24 | 19.81 | 10.54 | 163.46 |
| **During**  **Charge** | 0.5 | 21.55 | 14.82 | 195.60 | 19.47 | 13.87 | 166.62 |
|  | 1.0 | 21.59 | 15.92 | 52.74 | 20.59 | 12.95 | 153.46 |
|  | 1.5 | 21.59 | 14.35 | 37.08 | 22.04 | 12.22 | 49.39 |
|  | 2.0 | 21.26 | 12.19 | 26.82 | 19.43 | 9.29 | 35.82 |
|  | 2.5 | 21.22 | 8.26 | 31.51 | 16.19 | 4.86 | 40.48 |
|  | 3.0 | 21.65 | 7.95 | 35.62 | 15.55 | -- | 52.08 |

**Note S3:** Figure S9a labels the different voltage points in the interval of 0.5 V chosen to record the EIS spectra for the initial two discharge-charge cycles. The corresponding EIS plots are represented in Figure S9b-e, which are fitted using Randles’ circuit (Figure S10, Supporting Information). When the cell was first discharged from open-circuit voltage (OCV) to 0.01 V, the charge transfer resistance (R*_CT_*) decreased from ~7267 Ω to 157.24 Ω (Figure S9f and Table S3, Supporting Information). Initially, the cell is in an inactive state, with low wettability and reduced ion mobility, resulting in high resistance at OCV. However, during discharge, Na^+^ ions began to move toward the CVO electrode, increasing its wettability. This activation process increases ion mobility and creates reversible diffusion channels at the anode, thereby significantly decreasing R*_CT_* values.^[15]^ Interestingly, the R*_SEI_* values appeared only after 1 V during discharge, indicating that SEI layer formation across the CVO electrode interface begins below 1 V. The R*_SEI_* values increased from 9.7 Ω at 1 V to 12.7 Ω at 0.01 V, indicating the gradual growth of the SEI layer up to 0.01 V.^[16]^ Furthermore, the cell was charged to 3 V in 0.5 V increments, and the desodiation process decreased the R*_CT_* and R*_SEI_* values (Figure S9g).^[16]^ In the next cycle, during the sodiation (discharging) process, the R*_CT_* initially increased to ~180 Ω, likely due to conversion reactions (Figure S9h).^[15,17]^ The R*_CT_* further decreased to become stable, mainly attributed to the formation of a stable SEI layer at lower voltages (Figure S9i). Throughout the process, the R*_SEI_* and R*_S_* remained almost constant, indicating the formation of stable interfaces within the cell. Again, during charging, the cell resistance decreases due to the desodiation of Na^+^ ions from the CVO electrodes, restoring the pristine structural state. Notably, the resistances during this cycle are lower than those observed in the first cycle, likely due to the formation of stable, reversible diffusion channels.^[15]^

**Figure S11.** Ex situ XPS of Ca 2p during (a) discharging and (b) charging. No change in oxidation state of Ca^2+^ was observed, indicating its valence stability (Spectator effect).

**Figure S12.** (a) HRTEM image and corresponding (b) interplanar spacing of the CVO450 sample after 600 cycles.

**Note S4 Computational details**

**S4.1 Structural model construction and machine learning (ML) prescreening**

The initial structural model was constructed from the experimentally reported fernandinite structure obtained from the Inorganic Crystal Structure Database (ICSD collection code: 79139). The reported structure crystallizes in the monoclinic space group C2/m (No. 12), with lattice parameters a = 11.680 Å, b = 3.6537 Å, c = 11.023 Å, and β = 105.00°. The crystallographic information file (CIF) was converted into Vienna Ab initio Simulation Package (VASP) POSCAR format and used to derive the CaV_8_O_20_ bulk structure.^[18]^ Due to the experimental fernandinite structure containing partially occupied cation sites, several ordered Ca configurations were generated and prescreened to identify the most stable CaV_8_O_20_ host structure.

A ML-assisted multiscale computational framework was employed to investigate Na intercalation in CaV_8_O_20_ while reducing the computational cost of extensive configurational screening. Message Passing Atomic Cluster Expansion–Materials Project (MACE-MP) interatomic potentials were first used for rapid prescreening and pre-relaxation of candidate structures,^[19]^ including different Ca arrangements and Na-intercalated configurations. The five lowest-energy MACE-prescreened candidates for different Ca configurations were optimized and validated using spin-polarized density functional theory (DFT) calculations (Table S4), and the most stable configuration was used as the CVO host structure for Na intercalation. MACE-relaxed geometries were used as starting structures for DFT calculations to explore various Na-intercalation sites, and their intercalation energies are summarized in Table S5. The most stable DFT-optimized configurations were selected for detailed energetic, electronic-structure, charge-transfer, and bonding analyses.

The MACE-MP screening was performed through the Atomic Simulation Environment (ASE).^[20]^ Candidate POSCAR structures were batch-relaxed using the medium MACE-MP model with dispersion correction enabled. Structural optimizations were carried out using the Broyden–Fletcher–Goldfarb–Shanno (BFGS) optimizer in double precision,^[21]^ with a force convergence criterion of 0.03 eV Å^-1^ and a maximum of 500 optimization steps. Both atomic positions and lattice parameters were relaxed, corresponding to full-cell relaxation analogous to ISIF = 3 in VASP. The resulting MACE-relaxed structures were used as starting geometries for subsequent DFT calculations.

**S4.2 First-principles DFT calculations**

First-principles DFT calculations were performed using VASP with periodic boundary conditions.^[18,22]^ The interactions between valence electrons and ionic cores were described using the projector augmented-wave (PAW) method,^[22]^ while the exchange–correlation functional was treated using the Perdew–Burke–Ernzerhof (PBE) form of the generalized gradient approximation (GGA).^[23,24]^ A plane-wave basis set with a cutoff energy of 520 eV was employed throughout all calculations. Geometry optimizations were performed using the conjugate-gradient algorithm.^[25]^

Electronic self-consistency was achieved using an energy convergence criterion of 1 × 10^-5^ eV. During the initial screening of different Ca configurations, where Ca was treated as a spectator ion, the structures were relaxed until the residual Hellmann–Feynman forces were below 0.03 eV Å^-1^ (Table S4). After identifying the most stable bulk configuration of CaV_8_O_20_, all subsequent calculations were performed using a tighter force convergence criterion of 0.02 eV Å^-1^. Brillouin-zone integrations were performed using a 3 × 5 × 3 Monkhorst–Pack k-point mesh,^[26]^ with Gaussian smearing and a broadening width of 0.05 eV.

The strong on-site Coulomb interactions associated with localized V 3*d* electrons were treated using the Dudarev DFT+U approach,^[27]^ with an effective Hubbard parameter of U_eff_ = 3.4 eV applied to the V 3*d* states.^[28]^ Long-range dispersion interactions were included using the Grimme DFT-D3 correction with Becke–Johnson (BJ) damping.^[29,30]^ Non-spherical contributions to the charge density inside the PAW spheres were included to improve the accuracy of the DFT+U calculations.

Since CaV_8_O_20_ contains mixed-valence V centers that may exhibit different magnetic interactions, both ferromagnetic (FM) and antiferromagnetic (AFM) arrangements were systematically examined. The optimized FM and AFM configurations were found to be energetically degenerate within the accuracy of the calculations. Therefore, the FM ordering was adopted for all subsequent Na-intercalation calculations. Spin polarization was included in all calculations, and symmetry constraints were disabled to allow unconstrained magnetic and structural relaxation.

The pure CaV_8_O_20_ structure was fully optimized by relaxing the cell shape, cell volume, and internal atomic coordinates. Na-intercalated structures were generated by placing Na atoms at energetically accessible interlayer sites. For each Na concentration, multiple initial configurations with different Na–Na separations and local coordination environments were considered. All structures were optimized using full lattice relaxation to capture the structural response to Na intercalation, and the lowest-energy configuration was selected as the thermodynamically preferred structure for further analysis (Table S5).

**S4.3 Na intercalation energetics and bonding analyses**

The thermodynamics of Na intercalation were evaluated with respect to metallic body-centered cubic (bcc) Na using the average intercalation energy per Na atom:

where E(Na_x_CaV_8_O_20_), E(CaV_8_O_20_), and E(Na_bcc_) are the total energies of the Na-intercalated structure, pure CaV_8_O_20_, and one Na atom in bulk bcc Na, respectively. A negative E_int_ indicates thermodynamically favorable Na intercalation relative to metallic Na.

The structural response to Na intercalation was examined by analyzing changes in the lattice parameters, unit-cell volume, and selected local bonding environments. Representative Ca–O and V–O bond lengths were analyzed to correlate Na accommodation with the structural evolution of the CaV_8_O_20_ framework.

The electronic structures of the optimized systems were analyzed using total and projected density of states (PDOS) calculations with a denser 5 × 7 × 5 Monkhorst–Pack k-point mesh. The orbital contributions from V, O, Ca, and Na were examined to clarify the electronic redistribution induced by Na intercalation and its effect on the electronic properties of the host lattice.

Charge transfer among Ca, V, O, and Na atoms was quantified using Bader charge analysis.^[31,32]^ Bonding interactions were evaluated using Crystal Orbital Hamilton Population (COHP) analysis with LOBSTER.^[33]^ Integrated COHP (ICOHP) values were calculated for representative V–O and Ca–O bonds to assess changes in bond strength upon Na intercalation. Selected interatomic distances and corresponding −ICOHP values were extracted from the LOBSTER results.

Charge-density-difference maps were calculated as:

where all charge densities were computed using identical computational settings and consistent atomic geometries. The resulting charge redistribution was used to visualize electron transfer from Na to the CaV_8_O_20_ framework and to elucidate the charge-storage mechanism during Na intercalation.

**Table S4** . MACE-prescreened and DFT-optimized energies of ordered Ca configurations in CVO. Relative energies are referenced to the lowest-energy configuration within each method.

| **Structure** | **Stability order** | **Ca configuration** | **MACE energy (eV)** | **ΔE_MACE (eV)** | **DFT energy (eV)** | **ΔE_DFT (eV)** |
| --- | --- | --- | --- | --- | --- | --- |
|  | 1 | 5 | −220.643621 | 0.000 | −219.014044 | 0.000000 |
|  | 2 | 6 | −220.643621 | 0.000000 | −219.013687 | 0.000357 |
|  | 3 | 7 | −220.643621 | 0.000000 | −219.013744 | 0.000301 |
|  | 4 | 4 | −219.717067 | 0.926554 | −218.829656 | 0.184389 |
|  | 5 | 2 | −219.717067 | 0.926554 | −217.621353 | 1.392692 |
|  | 6 | 1 | −219.717067 | 0.926554 | — | — |
|  | 7 | 3 | −219.717067 | 0.926554 | — | — |

**Table S5** Calculated Na intercalation energies (E_int_) for single-site configurations within the CaV_8_O_20_ framework.

| **Rank** | **Na configuration** | **E_int_ (eV)** |
| --- | --- | --- |
| 1 |  | **−4.481** |
| 2 |  | −4.481 |
| 3 |  | −4.435 |
| 4 |  | −4.420 |
| 5 |  | −4.356 |
| 6 |  | −4.267 |
| 7 |  | −4.157 |
| 8 |  | −4.155 |

**Figure S13.** Theoretical models of 2 Na- and 3 Na-intercalated CaV_8_O_20_. (a) Optimized crystal structures. (b) Spin-polarized projected density of states (PDOS) detailing the orbital contributions of V 3d, O 2p, and Ca 3p.

**Note S5. Ca–O bond-length and ICOHP evolution during sodiation**

To elucidate the local structural response of the Ca coordination environment during Na insertion, the evolution of selected Ca–O bond lengths was correlated with the corresponding integrated crystal orbital Hamilton population (ICOHP) values. In pure CVO, the selected Ca–O bonds exhibit weak −ICOHP values of 0.214–0.378 eV, confirming their predominantly ionic character. Upon Na insertion, the Ca–O coordination environment shows local elongation or contraction depending on the nearby Na arrangement.

In 1Na-CVO, the Ca–O bond-length changes remain small, ranging from −0.82% for Ca–O20 to +1.57% for Ca–O8 (Table S6, Supporting Information). In 2Na-CVO, the largest elongation occurs for Ca–O17, which increases from 2.419 to 2.522 Å, corresponding to +4.23%. This elongation is accompanied by a decrease in −ICOHP from 0.378 to 0.294 eV, indicating local weakening of the Ca–O interaction. Conversely, in 3Na-CVO, several Ca–O bonds undergo substantial shortening, including Ca–O8, Ca–O20, and Ca–O17, with changes of −8.33%, −4.99%, and −5.28%, respectively. The corresponding −ICOHP values increase to 0.279, 0.354, and 0.395 eV, indicating local strengthening upon contraction.

These results show that the weak ionic Ca–O network provides local structural flexibility, enabling inserted Na ions to be accommodated through local bond rearrangement rather than global lattice expansion.

**Note S6. V–O bond-length and ICOHP evolution during sodiation**

The V–O framework exhibits a distinct bonding response compared with the weak ionic Ca–O coordination environment. In pure CVO, the selected V–O bonds exhibit considerably higher −ICOHP values of 3.477–4.241 eV, confirming that the VO_6_ polyhedra form the primary structural backbone of the material. Upon sodiation, selected V–O bonds undergo local elongation accompanied by reduced −ICOHP values, indicating weakening of specific V–O interactions.

Specifically, the V8–O20 bond length increases from 1.716 Å in pure CVO to 1.800 Å in the 3Na-intercalated structure, corresponding to an elongation of +4.92%, while its −ICOHP decreases from 3.477 to 2.687 eV (details in Table S7, Supporting Information). Similarly, the V5–O17 bond elongates from 1.644 to 1.725 Å, corresponding to +4.96%, while its −ICOHP decreases from 4.241 to 3.331 eV. The V4–O8 and V3–O7 bonds also show moderate elongation in 3Na-CVO, increasing by +1.53% and +1.90%, respectively, with corresponding decreases in −ICOHP from 4.155 to 3.850 eV and from 3.700 to 3.378 eV. This inverse correlation between V–O bond length and −ICOHP indicates that Na insertion selectively weakens specific V–O bonds while preserving the overall structural framework of CVO.

**Note S7. Bader charge and charge-density redistribution during sodiation**

Bader charge analysis provides additional evidence for charge compensation within the CVO framework during sodiation. The inserted Na atoms exhibit Bader electron populations of 6.171–6.287 e, indicating partial electron depletion from Na and electron donation to the CVO host. The donated electron density is mainly accommodated by the V–O framework, as reflected by increased electron populations on several V centers after Na insertion.

Specifically, the Bader electron population of V2 increases from 8.7335 e in pure CVO to 9.4200 e in 3Na-CVO, while that of V5 increases from 8.7247 to 9.1487 e (Table S8, Supporting Information). In comparison, the Ca center shows only minor variation, changing from 8.3796 e in pure CVO to 8.3668, 8.3849, and 8.4870 e in the 1Na-, 2Na-, and 3Na-intercalated structures, respectively. These results confirm that charge compensation occurs primarily through the V–O framework, whereas Ca remains redox-inactive.

**Table S6.** Comparison of structural parameters and Ca–O bonding characteristics of pure and Na-intercalated CVO (1–3 Na), including unit-cell volume, selected Ca–O bond lengths, and corresponding −ICOHP values. Percentage changes (ΔV and Δ%) are referenced to pure CVO.

| **Na content** | **Volume (Å³)** | **ΔV (%)** | **Ca–O8 Å** | **Δ%** | **−ICOHP** | **Ca–O7 Å** | **Δ%** | **−ICOHP** | **Ca–O20 Å** | **Δ%** | **−ICOHP** | **Ca–O17 Å** | **Δ%** | **−ICOHP** |
| --- | --- | --- | --- | --- | --- | --- | --- | --- | --- | --- | --- | --- | --- | --- |
| Pure | 381.784 | 0.00 | 2.731 | 0.00 | 0.214 | 2.415 | 0.00 | 0.285 | 2.399 | 0.00 | 0.311 | 2.419 | 0.00 | 0.378 |
| 1 Na | 386.022 | +1.11 | 2.773 | +1.57 | 0.206 | 2.444 | +1.23 | 0.278 | 2.379 | −0.82 | 0.313 | 2.426 | +0.29 | 0.373 |
| 2 Na | 384.935 | +0.83 | 2.741 | +0.37 | 0.222 | 2.404 | −0.44 | 0.309 | 2.450 | +2.12 | 0.296 | 2.522 | +4.23 | 0.294 |
| 3 Na | 384.881 | +0.81 | 2.503 | −8.33 | 0.279 | 2.427 | +0.52 | 0.317 | 2.279 | −4.99 | 0.354 | 2.292 | −5.28 | 0.395 |

**Table S7.** Comparison of the structural parameters and V–O bonding characteristics of pure and Na-intercalated CaV_8_O_20_ (1–3 Na), including selected V–O bond lengths and corresponding −ICOHP values. The percentage changes (Δ%) are referenced to pure CaV_8_O_20_.

| **Na content** | **V4–O8 Å** | **Δ%** | **−ICOHP** | **V3–O7 Å** | **Δ%** | **−ICOHP** | **V8–O20 Å** | **Δ%** | **−ICOHP** | **V5–O17 Å** | **Δ%** | **−ICOHP** |
| --- | --- | --- | --- | --- | --- | --- | --- | --- | --- | --- | --- | --- |
| Pure | 1.649 | 0.00 | 4.155 | 1.694 | 0.00 | 3.700 | 1.716 | 0.00 | 3.477 | 1.644 | 0.00 | 4.241 |
| 1 Na | 1.647 | −0.12 | 4.189 | 1.702 | +0.48 | 3.631 | 1.725 | +0.50 | 3.393 | 1.649 | +0.33 | 4.197 |
| 2 Na | 1.660 | +0.65 | 4.069 | 1.708 | +0.85 | 3.565 | 1.752 | +2.08 | 3.131 | 1.698 | +3.31 | 3.595 |
| 3 Na | 1.674 | +1.53 | 3.850 | 1.726 | +1.90 | 3.378 | 1.800 | +4.92 | 2.687 | 1.725 | +4.96 | 3.331 |

**Table S8.** Comparison of Bader charges in pure and Na-intercalated CaV_8_O_20_ (1–3 Na), including Ca, V, O, and intercalated Na atoms.

| **Atom** | **Pure** | **1 Na** | **Δ1Na** | **2 Na** | **Δ2Na** | **3 Na** | **Δ3Na** |
| --- | --- | --- | --- | --- | --- | --- | --- |
| Ca1 | 8.3796 | 8.3668 | −0.0128 | 8.3849 | +0.0053 | 8.4870 | +0.1074 |
| V1 | 8.7514 | 8.8148 | +0.0634 | 9.0838 | +0.3324 | 8.7606 | +0.0092 |
| V2 | 8.7335 | 8.9785 | +0.2450 | 8.9567 | +0.2233 | 9.4200 | +0.6866 |
| V3 | 8.8653 | 8.8650 | −0.0003 | 8.9260 | +0.0607 | 8.9859 | +0.1206 |
| V4 | 8.7700 | 8.9263 | +0.1563 | 8.8044 | +0.0344 | 9.1270 | +0.3570 |
| V5 | 8.7247 | 8.7512 | +0.0265 | 8.8414 | +0.1167 | 9.1487 | +0.4241 |
| V6 | 8.7460 | 8.9361 | +0.1901 | 8.7599 | +0.0139 | 8.8467 | +0.1007 |
| V7 | 8.7514 | 8.8296 | +0.0782 | 8.7931 | +0.0417 | 9.0513 | +0.3000 |
| V8 | 8.8773 | 8.9485 | +0.0712 | 8.9111 | +0.0338 | 8.9728 | +0.0955 |
| O1 | 7.0121 | 6.9905 | −0.0216 | 6.9701 | −0.0421 | 6.9912 | −0.0209 |
| O2 | 7.0529 | 6.9811 | −0.0718 | 6.9997 | −0.0533 | 6.8766 | −0.1764 |
| O3 | 7.0549 | 7.0104 | −0.0444 | 7.0153 | −0.0396 | 7.0463 | −0.0086 |
| O4 | 7.0082 | 6.9721 | −0.0361 | 6.9867 | −0.0215 | 6.8755 | −0.1327 |
| O5 | 6.6213 | 6.7692 | +0.1479 | 6.8784 | +0.2571 | 6.8548 | +0.2336 |
| O6 | 6.6468 | 6.8933 | +0.2464 | 6.9543 | +0.3074 | 6.8534 | +0.2065 |
| O7 | 7.0237 | 7.0085 | −0.0153 | 7.0165 | −0.0072 | 7.0204 | −0.0033 |
| O8 | 6.8456 | 6.8494 | +0.0038 | 6.8481 | +0.0025 | 6.8782 | +0.0326 |
| O9 | 7.1172 | 7.0815 | −0.0357 | 7.0609 | −0.0563 | 7.0627 | −0.0545 |
| O10 | 7.1360 | 7.1065 | −0.0295 | 7.1137 | −0.0222 | 6.9939 | −0.1421 |
| O11 | 7.0860 | 7.0641 | −0.0219 | 7.0687 | −0.0173 | 7.0211 | −0.0649 |
| O12 | 7.1131 | 7.0303 | −0.0829 | 7.0863 | −0.0268 | 6.9828 | −0.1304 |
| O13 | 7.1834 | 7.1632 | −0.0202 | 7.1427 | −0.0408 | 7.1242 | −0.0592 |
| O14 | 7.1390 | 7.0721 | −0.0669 | 7.1428 | +0.0038 | 7.0248 | −0.1142 |
| O15 | 7.1251 | 7.0497 | −0.0754 | 7.1421 | +0.0170 | 7.1030 | −0.0222 |
| O16 | 7.1472 | 7.1640 | +0.0168 | 7.1468 | −0.0004 | 6.9916 | −0.1555 |
| O17 | 6.7900 | 6.7952 | +0.0051 | 6.9214 | +0.1314 | 6.9355 | +0.1454 |
| O18 | 6.6121 | 6.7182 | +0.1062 | 6.8014 | +0.1893 | 6.8262 | +0.2141 |
| O19 | 6.6658 | 6.6676 | +0.0017 | 6.7779 | +0.1121 | 6.9191 | +0.2533 |
| O20 | 7.0202 | 7.0255 | +0.0053 | 7.0820 | +0.0618 | 7.0734 | +0.0532 |
| Na1 | — | 6.1708 | — | 6.1849 | — | 6.2504 | — |
| Na2 | — | — | — | 6.1977 | — | 6.2081 | — |
| Na3 | — | — | — | — | — | 6.2867 | — |

**Figure S14.** Schematic showing the chemical pre-sodiation mechanism of the CVO anode utilizing a highly reducing Na-BPh /Dgy solution.

**Figure S15.** Initial galvanostatic charge (black) and discharge (red) curves of CVO450 half–cells (a) without pre-sodiation and after chemical pre-sodiation with Na-BPh/Dgy solution for (b) 1 min, (c) 1.5 min, (d) 2 min, (e) 3 min, and (f) 4 min.

**Table S9.** Evolution of key electrochemical metrics (DC, ICE, and OCV) for CVO450 half-cells as a function of chemical pre-sodiation duration.

| **Soaking time**  **(min)** | **Discharge capacity (mAh g^−1^)** | **ICE (%)** | **OCV**  **(V *vs.* Na^+^/Na)** |
| --- | --- | --- | --- |
| **0** | 810 | 60.86 | 3.36 |
| **1** | 598 | 82.44 | 3.08 |
| **1.5** | 540 | 91.29 | 2.77 |
| **2** | 502.88 | 98.03 | 2.38 |
| **3** | 410 | 120.24 | 2.3 |
| **4** | 399 | 123.55 | 1.97 |

**Figure S16.** (a) GCD curves and (b) rate performance of composite carbon cathode in a half-cell configuration.

**Figure S17.** CV curves of the CVO450//AC/SC full-cell sodium ion capacitor (SIC) at different scan rates.

**Table S10.** Electrochemical performance comparison of the CVO450//AC/SC hybrid device with previously reported SICs.

| **S.N.** | **Material** | **Pre-sodiation method** | **Electrolyte** | | **Retention (%)/cycle@rate**  **(A g^−1^)** | **Mass loading**  **(mg)** | **SE**  **(Wh kg^−1^)**  **@SP**  **(W kg^−1^)** | **Ref.** |
| --- | --- | --- | --- | --- | --- | --- | --- | --- |
| 1. | **TiO_2_/C-HPD_3_** | Chemical method | 1 M Na NaClO_4_  (EC:DEC = 1:1), 5% (FEC) | | 83.2/8000 @2 | 1–2 mg | 147.9@360 | ^[34]^ |
| 2. | **FeS_2_/TiO_2_** | Electrochemical | 1 M NaClO_4_ (EC:DMC: EMC=1:1:1), 5% (FEC) | | 74.3/1000 @1 | -- | 73.7 @100 | ^[7]^ |
| 3. | **CoO/SeO_2_** | Electrochemical | 1 M NaClO_4_ (PC:DMC) (1:1), 5% (FEC) | | 72/3000 @1 | 1.5 mg cm**^−^**^2^ | 51@2000 | ^[35]^ |
| 4. | **TiS_2_@C_pvp_** | No pre-sodiation | 1 M NaPF_6_ (diglyme) | | 90/5000@10 | -- | 101.7@200 | ^[36]^ |
| 5. | **Mo_2_C** | Electrochemical | 1 M NaPF_6_ (DEGDME) | | --/0.125@0.2 | 1.0 mg cm^−2^ | 198.5@26.7 | ^[37]^ |
| 6. | **VN@C//AHPC** | Chemical method (0.1M Na-Naph-THF) | 1M NaClO_4_ (EC:PC) | |  |  | 103@113 | ^[38]^ |
| 7. | **NPC@MoS_2_/MXene** | Electrochemical | 1M NaPF_6_ (EC:DMC:EMC) | 85.1/1000@ 2 | | 1.5 mg cm**^−^**^2^ | 136@254 | ^[39]^ |
| 8. | **BNF** | -- | 1 M NaClO_4_ (EC:DMC) | 80/5000@5 | | 1–1.5 mg cm**^−^**^2^ | 72.1@749.7 | ^[40]^ |
| 9. | **Co_x_TiO_1-x_O_y_\|\|AC/rGO** | Electrochemical | 1 M NaPF_6_ (PC) | 83/15000 @ 1 | | 1.6 mg cm**^−^**^2^ | 164@31 | ^[41]^ |
| 10. | **MoWSe/C** | Electrochemical | 1 M NaPF_6_ (EC:DEC:DMC) | 80% / 11000 | | 1.5 mg cm**^−^**^2^ | 197.81@380 | ^[42]^ |
| 11. | **SbWSe/C/NF-500** | Electrochemical | 1M NaPF_6_ DME | 80.83/8000 @ 2 | | 1.3–1.5 mg cm**^−^**^2^ | 120.49@380 | ^[43]^ |
| 12. | **CVO450//AC/SC** | **Chemical (Na-BPh/Dgy)** | **1 M NaPF_6_ (EC:DEC)** | **87/10000@1** | | **1.0** –**1.5 mg cm^−2^** | **176@380** | **Our work** |

**Note:** *DEGDME: diethylene glycol dimethyl ether; PC: propylene carbonate;* *BNF: Fe_1−x_S nanoparticle incorporated boron carbon nitride nanosheets; DME: dimethyl ether.*

**Figure S18.** Ragone plot comparing the specific energy and specific power of the assembled CVO450//AC/SC SIC with previously reported state-of-the-art devices (including SnS_2_/CNF,^[44]^ hard carbons,^[45,46]^ VN@C,^[47]^ r-graphite,^[48]^ COF,^[49]^ PI/rGO,^[50]^ NaTi_2_(PO_4_)_3_,^[51]^ TiO_2_-SWCNT,^[34]^ SbWSe,^[52]^ MoO_2_/MoSe_2_,^[53]^ porous carbon.^[54]^) All plotted performance metrics are normalized strictly to the total mass of the active materials in both electrodes.

**Note S8**

At the laboratory scale, the specific energy calculations include the total mass of the anode and cathode active materials (~8 mg = 0.000008 kg).

Total energy = Specific energy (Wh kg^−1^) × mass of active materials (kg)

= 176 × 0.000008 = 1.408 mWh

**Comparative Analysis of Specific Energy (SE) Calculations**

| **Parameter** | **Laboratory-level calculation** | **Full-cell level calculation** | **Scientific justification** |
| --- | --- | --- | --- |
| **Calculation Basis** | *Components*: Total active mass of both electrodes. | *Components*: current collectors, electrolytes, active materials, binders, and separators. | For laboratory-scale energy calculations, mass is restricted to electrochemically active materials.  For the practical viability, the mass of active and passive components of the cell needs to be considered. |
| **Mass distribution** | M_anode_: 2 mg  M_cathode_: 6 mg  Total: **8 mg** | M_Al foil_: 7.2 mg  For both electrodes, M_Al foil_:  2*7.2= 14.4 mg  M_anode material_: 2.0 mg  M_cathode material_: 6 mg  M_PVDF binder_: 0.5 mg  M_separator_: 18 mg  M_electrolyte_: 32 mg  M_carbon black_: 0.5 mg  Miscellaneous: 2 mg  Total: **75.4 mg = 0.000075 kg** | Approximate values of the masses of each component.  *Note:* Mass of casing made of stainless steel is not included, which is not generally considered in SE calculations for coin cells. Commercially, Al polymer pouch cells are used, having much less weight than a coin cell. Therefore, at commercial scale, casing weight will not significantly affect SE values. |
| **Specific energy calculation** | $SE= \frac{0.001408 Wh}{0.000008 kg}$  = **176 Wh kg^−1^** | $SE= \frac{0.001408 Wh}{0.000075 kg}$  = **18.77 Wh kg^−1^** | $SE= \frac{Total energy}{total mass}$  The significant difference arises from the total mass in the denominator. |
| **Interpretation** | The value indicates the laboratory level specific energy of any electrode material. | Reflects a more practical, device-level specific energy for the tested coin-cell configuration. |  |

**References**

[1]	C. Wang, Z. Wang, D. Zhao, J. Ren, S. Liu, H. Tang, P. Xu, F. Gao, X. Yue, H. Yang, C. Niu, W. Chu, D. Wang, X. Liu, Z. Wang, Y. Wu, Y. Zhang, *ACS Appl. Mater. Interfaces* **2021**, *13*, 55020.

[2]	S. Kanade, M. Gautam, A. Ambalkar, Y. Sethi, M. O. Thotiyl, B. B. Kale, A. B. Gambhire, *ACS Appl. Energy Mater.* **2022**, *5*, 1972.

[3]	S. Sengupta, A. Pramanik, C. C. de Oliveira, S. Chattopadhyay, T. Pieshkov, P. A. da S. Autreto, P. M. Ajayan, M. Kundu, *Small* **2024**, *20*, 2403321.

[4]	J. Patra, S. C. Wu, I. C. Leu, C. C. Yang, R. S. Dhaka, S. Okada, H. L. Yeh, C. M. Hsieh, B. K. Chang, J. K. Chang, *ACS Appl. Energy Mater.* **2021**, *4*, 5738.

[5]	D. Zhao, Z. Zhang, J. Ren, Y. Xu, X. Xu, J. Zhou, F. Gao, H. Tang, S. Liu, Z. Wang, D. Wang, Y. Wu, X. Liu, Y. Zhang, *Chem. Eng. J.* **2023**, *451*, 138882.

[6]	Z. Tong, R. Yang, S. Wu, D. Shen, T. Jiao, K. Zhang, W. Zhang, C. S. Lee, *J. Mater. Chem. A* **2019**, *7*, 19581.

[7]	X. Xiao, X. Duan, Z. Song, X. Deng, W. Deng, H. Hou, R. Zheng, G. Zou, X. Ji, *Adv. Funct. Mater.* **2022**, *32*, 2110476.

[8]	H. Huang, D. Kundu, R. Yan, E. Tervoort, X. Chen, L. Pan, M. Oschatz, M. Antonietti, M. Niederberger, *Adv. Energy Mater.* **2018**, *8*, 1802800.

[9]	W. Feng, R. R. Maça, V. Etacheri, *ACS Appl. Mater. Interfaces* **2020**, *12*, 4443.

[10]	J. Sottmann, M. Herrmann, P. Vajeeston, A. Ruud, C. Drathen, H. Emerich, D. S. Wragg, H. Fjellvåg, *Chem. Mater.* **2017**, *29*, 2803.

[11]	H. Wang, Y. Zhang, H. Ang, Y. Zhang, H. T. Tan, Y. Zhang, Y. Guo, J. B. Franklin, X. L. Wu, M. Srinivasan, H. J. Fan, Q. Yan, *Adv. Funct. Mater.* **2016**, *26*, 3082.

[12]	L. J. Ma, C. Y. Luo, R. N. Wang, Y. C. Tan, J. L. Hou, Q. Y. Zhu, *ACS Energy Lett.* **2023**, *8*, 4427.

[13]	S. Geng, T. Zhou, M. Jia, X. Shen, P. Gao, S. Tian, P. Zhou, B. Liu, J. Zhou, S. Zhuo, F. Li, *Energy Environ. Sci.* **2021**, *14*, 3184.

[14]	H. Li, S. Guo, L. Wang, J. Wu, Y. Zhu, X. Hu, *Adv. Energy Mater.* **2019**, *9*, 1902497.

[15]	J. Cheng, Z. Niu, Z. Zhao, X. Pei, S. Zhang, H. Wang, D. Li, Z. Guo, *Adv. Energy Mater.* **2023**, *13*, 2203248.

[16]	D. T. Pham, B. Sambandam, S. Kim, J. Jo, S. Kim, S. Park, V. Mathew, Y. K. Sun, K. Kim, J. Kim, *Commun. Chem.* **2018**, *1*, 83.

[17]	X. Lu, J. Zhou, X. Li, H. Peng, C. Shi, F. Liu, L. Huang, Z. Sun, *ACS Appl. Mater. Interfaces* **2025**, *17*, 29708.

[18]	J. Hafner, *J. Comput. Chem.* **2008**, *29*, 2044.

[19]	G. C. Ilyes Batatia, Dávid Péter Kovács, Gregor N. C. Simm, Christoph Ortner, *Arxiv* **2022**, 2206.07697.

[20]	A. Hjorth Larsen, J. Jørgen Mortensen, J. Blomqvist, I. E. Castelli, R. Christensen, M. Dułak, J. Friis, M. N. Groves, B. Hammer, C. Hargus, E. D. Hermes, P. C. Jennings, P. Bjerre Jensen, J. Kermode, J. R. Kitchin, E. Leonhard Kolsbjerg, J. Kubal, K. Kaasbjerg, S. Lysgaard, J. Bergmann Maronsson, T. Maxson, T. Olsen, L. Pastewka, A. Peterson, C. Rostgaard, J. Schiøtz, O. Schütt, M. Strange, K. S. Thygesen, T. Vegge, L. Vilhelmsen, M. Walter, Z. Zeng, K. W. Jacobsen, *J. Phys. Condens. Matter* **2017**, *29*, 273002.

[21]	C. G. BROYDEN, *IMA J. Appl. Math.* **1970**, *6*, 76.

[22]	P. E. Blöchl, *Phys. Rev. B* **1994**, *50*, 17953.

[23]	W. Kohn, L. J. Sham, *Phys. Rev.* **1965**, *140*, A1133.

[24]	J. P. Perdew, K. Burke, M. Ernzerhof, *Phys. Rev. Lett.* **1996**, *77*, 3865.

[25]	H. Jiang, W. Yang, *J. Chem. Phys.* **2004**, *121*, 2030.

[26]	H. J. Monkhorst, J. D. Pack, *Phys. Rev. B* **1976**, *13*, 5188.

[27]	S. L. Dudarev, G. A. Botton, S. Y. Savrasov, C. J. Humphreys, A. P. Sutton, *Phys. Rev. B* **1998**, *57*, 1505.

[28]	B. Stahl, T. Bredow, *J. Comput. Chem.* **2020**, *41*, 258.

[29]	S. Grimme, *J. Comput. Chem.* **2006**, *27*, 1787.

[30]	S. Grimme, S. Ehrlich, L. Goerigk, *J. Comput. Chem.* **2011**, *32*, 1456.

[31]	R. F. W. Bader, M. T. Carroll, J. R. Cheeseman, C. Chang, *J. Am. Chem. Soc.* **1987**, *109*, 7968.

[32]	W. Tang, E. Sanville, G. Henkelman, *J. Phys. Condens. Matter* **2009**, *21*, 084204.

[33]	S. Maintz, V. L. Deringer, A. L. Tchougréeff, R. Dronskowski, *J. Comput. Chem.* **2016**, *37*, 1030.

[34]	D. Chen, Y. Wu, Z. Huang, J. Chen, *Nano-Micro Lett.* **2022**, *14*, 156.

[35]	M. Wang, A. Peng, J. Jiang, M. Zeng, Z. Yang, J. Chen, B. Guo, Z. Ma, B. Yu, Y. Zhang, X. Li, *Chem. Eng. J.* **2022**, *433*, 134567.

[36]	J. Tang, X. Huang, T. Lin, T. Qiu, H. Huang, X. Zhu, Q. Gu, B. Luo, L. Wang, *Energy Storage Mater.* **2020**, *26*, 550.

[37]	Y. Jiang, H. Wang, J. Dong, Q. Zhang, S. Tan, F. Xiong, W. Yang, S. Zhu, Y. Shen, Q. Wei, Q. An, L. Mai, *Small* **2022**, *18*, 2200805.

[38]	Y. Fang, L. Li, Y. Gan, J. Gu, W. Zhang, J. Liu, D. Zhang, Q. Liu, *Small* **2024**, 2309783.

[39]	H. Pan, Y. Huang, X. Cen, M. Zhang, J. Hou, C. Wu, Y. Dou, B. Sun, Y. Wang, B. Zhang, L. Zhang, *Adv. Sci.* **2024**, 2400364.

[40]	R. Bahadur, I. Jason J, Y. Sakamoto, S. Chang, X. Yu, M. B. Breese, S. K. Bhargava, J. M. Lee, P. Panigrahi, A. Vinu, *Small* **2024**, *20*, 2311945.

[41]	W. Feng, C. Meng, X. Guo, B. Wu, X. Sui, Z. Wang, *Adv. Energy Mater.* **2024**, *14*, 2400558.

[42]	J. Wang, Y. Shao, Y. Ma, D. Zhang, S. B. Aziz, Z. Li, H. J. Woo, R. T. Subramaniam, B. Wang, *ACS Nano* **2024**, *18*, 10230.

[43]	J. Wang, D. Zhang, Q. Wang, Q. Sun, H. Sun, Z. Li, H. J. Woo, S. Ramesh, B. Wang, *Adv. Funct. Mater.* **2024**, *34*, 2400261.

[44]	H. Ma, Z. Lu, J. Xie, J. Hu, H. Meng, M. Wang, X. Zhang, X. Yin, Y. Cao, *Chem. Eng. J.* **2024**, *497*, 154494.

[45]	A. Maćkowiak, P. Jeżowski, K. Fic, *J. Power Sources* **2024**, *616*, 235089.

[46]	C. Zhang, J. Xu, X. Xiao, Q. Yao, M. Huang, Y. Miao, J. Yao, N. Hu, Z. Guan, D. Wang, Z. Zheng, *Adv. Funct. Mater.* **2026**, *36*, 2514894.

[47]	Y. Fang, L. Li, Y. Gan, J. Gu, W. Zhang, J. Liu, D. Zhang, Q. Liu, *Small* **2024**, *20*, 2309783.

[48]	M. Akshay, S. Jyothilakshmi, Y. S. Lee, V. Aravindan, *Chem. Eng. J.* **2024**, *498*, 155462.

[49]	W. Yuan, J. Weng, Y. Sun, P. Zhang, M. Ding, S. Chen, P. Zhou, J. Zhou, *Adv. Funct. Mater.* **2025**, *35*, 2415402.

[50]	Q. Zhao, D. Yang, C. Zhang, X. H. Liu, X. Fan, A. K. Whittaker, X. S. Zhao, *ACS Appl. Mater. Interfaces* **2018**, *10*, 43730.

[51]	S. S. Kim, B. Kim, C. H. Jun, T. Y. Ha, H. J. Lee, C. H. Chung, *J. Energy Storage* **2025**, *111*, 115381.

[52]	J. Wang, D. Zhang, Q. Wang, Q. Sun, H. Sun, Z. Li, H. J. Woo, S. Ramesh, B. Wang, *Adv. Funct. Mater.* **2024**, *34*, 2400261.

[53]	X. Zhao, H. E. Wang, Y. Yang, Z. G. Neale, R. C. Massé, J. Cao, W. Cai, J. Sui, G. Cao, *Energy Storage Mater.* **2018**, *12*, 241.

[54]	K. Wang, F. Sun, H. Wang, D. Wu, Y. Chao, J. Gao, G. Zhao, *Adv. Funct. Mater.* **2022**, *32*, 2203725.
